# Lethal co-expression intolerance underlies the mutually exclusive expression of ASCL1 and NEUROD1 in SCLC cells

**DOI:** 10.1038/s41698-025-00860-6

**Published:** 2025-03-13

**Authors:** Hirofumi Watanabe, Yusuke Inoue, Kazuo Tsuchiya, Kazuhiro Asada, Makoto Suzuki, Hiroshi Ogawa, Masayuki Tanahashi, Takuya Watanabe, Shun Matsuura, Kazuyo Yasuda, Ippei Ohnishi, Shiro Imokawa, Hideki Yasui, Masato Karayama, Yuzo Suzuki, Hironao Hozumi, Kazuki Furuhashi, Noriyuki Enomoto, Tomoyuki Fujisawa, Kazuhito Funai, Kazuya Shinmura, Haruhiko Sugimura, Naoki Inui, Takafumi Suda

**Affiliations:** 1 Second Division, Department of Internal Medicine, Hamamatsu University School of Medicine, Hamamatsu, Japan; 2 Department of Tumor Pathology, Hamamatsu University School of Medicine, Hamamatsu, Japan; 3 Department of Respiratory Medicine, Shizuoka General Hospital, Shizuoka, Japan; 4 Department of Pathology, Shizuoka General Hospital, Shizuoka, Japan; 5 Department of Pathology, Seirei Mikatahara General Hospital, Hamamatsu, Japan; 6 Division of Thoracic Surgery, Respiratory Disease Center, Seirei Mikatahara General Hospital, Hamamatsu, Japan; 7 Department of Respiratory Medicine, Fujieda Municipal General Hospital, Fujieda, Japan; 8 Department of Pathology, Fujieda Municipal General Hospital, Fujieda, Japan; 9 Division of Pathology, Iwata City Hospital, Iwata, Japan; 10 Department of Respiratory Medicine, Iwata City Hospital, Iwata, Japan; 11 Department of Chemotherapy, Hamamatsu University School of Medicine, Hamamatsu, Japan; 12 First Department of Surgery, Hamamatsu University School of Medicine, Hamamatsu, Japan; 13 Sasaki Institute, Sasaki Foundation, Tokyo, Japan; 14 Department of Clinical Pharmacology and Therapeutics, Hamamatsu University School of Medicine, Hamamatsu, Japan

**Keywords:** Small-cell lung cancer, Molecular medicine

## Abstract

Small cell lung cancer (SCLC) subtypes, defined by the expression of lineage-specific transcription factors (TFs), are thought to be mutually exclusive, with intra-tumoral heterogeneities. This study investigated the mechanism underlying this phenomenon with the aim of identifying a novel vulnerability of SCLC. We profiled the expression status of ASCL1, NEUROD1, POU2F3, and YAP1 in 151 surgically obtained human SCLC samples. On subtyping, a high degree of mutual exclusivity was observed between ASCL1 and NEUROD1 expression at the cell, but not tissue, level. Inducible co-expression models of all combinations of ASCL1, NEUROD1, POU2F3, YAP1, and ATOH1 using SCLC cell lines showed that some expression combinations, such as ASCL1 and NEUROD1, exhibited mutual repression and caused growth inhibition and apoptosis. Gene expression and ATAC-seq analyses of the ASCL1 and NEUROD1 co-expression models revealed that co-expression of ASCL1 in NEUROD1-driven cells, and of NEUROD1 in ASCL1-driven cells, both (although more efficiently by the former) reprogrammed the cell lineage to favor the ectopically expressed factor, with rewiring of chromatin accessibility. Mechanistically, co-expressed NEUROD1 in ASCL1-driven SCLC cells caused apoptosis by downregulating BCL2, likely in a MYC-independent manner. In conclusion, lethal co-expression intolerance underlies the mutual exclusivity between these pioneer TFs, ASCL1 and NEUROD1, in an SCLC cell. Further investigation is warranted to enable therapeutic targeting of this vulnerability.

## Introduction

Small cell lung cancer (SCLC) is one of the deadliest human cancers^1^, accounting for up to 15% of all lung cancer cases. Unlike non-small cell lung cancer (NSCLC), for which targeting driver genetic alterations has achieved substantial success, the treatment strategy for SCLC has not changed dramatically for decades, mainly because of the absence of such druggable driver mutations. Furthermore, the addition of immune checkpoint blockade therapy to conventional platinum-based chemotherapy has shown limited benefits for the treatment of SCLC^2,3^ compared with NSCLC. As a result, the prognosis of SCLC patients remains dismal, urgently calling for translational and clinical progress for this recalcitrant cancer.

In addition to the unique molecular characteristics of SCLC, including the frequent loss of Rb and p53^1,4^, SCLC can be categorized based on the expression of several key lineage-specific transcription factors (TFs), including ASCL1 (achaete-scute homolog 1; SCLC-A), NEUROD1 (neurogenic differentiation factor 1; SCLC-N), and POU2F3 (POU class 2 homeobox 3; SCLC-P), alongside other putative TFs, YAP1 (yes-associated protein 1; SCLC-Y)^5,6^ and ATOH1 (atonal basic helix-loop-helix [bHLH] transcription factor 1)^7^. In general, the expression of these lineage-specific TFs in SCLC tumors is thought to be mutually exclusive or predominant for one TF^5,8^, with intra-tumoral heterogeneities^9^ and potential for plasticity between subtypes^10^, particularly subtype switching from SCLC-A to SCLC-N^11–15^.

Mutual exclusivity in cancer biology could offer an important clue for the discovery of previously unidentified vulnerabilities and therapeutic strategies. A remarkable example is the strong mutually exclusive mutation pattern between the most common driver mutations in lung adenocarcinoma, the *EGFR* and *KRAS* mutations^16^. Although this has been described as resulting from functional redundancy in oncogenic pathway activation^17^, this could also be explained by an alternative mechanism that the level of oncogenic signaling through the MAPK pathway—and thus the level of activation of ERK—in oncogene-addicted cancer cells requires a fine balance, and that an overdose of oncogenic signaling could be toxic^18,19^. Of clinical relevance, this vulnerability has the potential to be exploited as a novel strategy to treat cancers harboring ERK-activating alterations by the therapeutic overactivation of oncogenic signaling^20,21^.

We hypothesized that some combinations of lineage-specific TFs may cause a conflict and survival disadvantage when co-expressed in SCLC cells, based on reports of the mutually exclusive expression of lineage-specific TFs in SCLC tumors and preferential subtype switching rather than the co-expression of two or more TFs^11–15^. In this study, we aimed to identify the TF co-expression patterns that exert detrimental effects in SCLC cells. By uncovering the mechanisms underlying this lineage-specific intolerance to the co-expression of another TF, we also aimed to provide new insights into the understanding of SCLC biology and reveal a novel vulnerability of this disease.

## Results

### Mutual exclusivity of lineage-specific TFs in clinical SCLC tumors

**Fig. 1:**
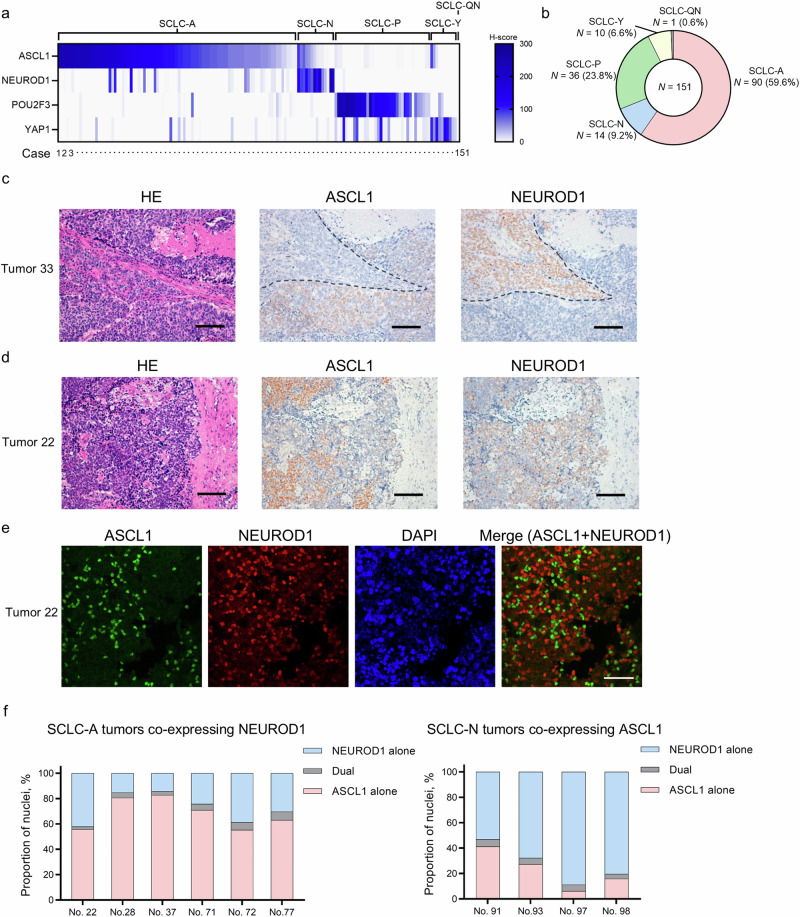
Expression profiling of lineage-specific transcription factors in clinical SCLC tumors. **a** Heatmap showing the expression of ASCL1, NEUROD1, POU2F3, and YAP1 in clinical SCLC tumors. A total of 151 SCLC tumors were classified as ASCL1-dominant (SCLC-A), NEUROD1-dominant (SCLC-N), POU2F3-dominant (SCLC-P), YAP1-dominant (SCLC-Y), or quadruple-negative (SCLC-QN), based on the predominantly expressed transcription factor. **b** Proportions of the SCLC molecular subtypes in the 151 tumors. **c** Representative images of the spatially separated expression patterns of ASCL1 and NEUROD1 by immunohistochemical staining in consecutive sections of Tumor 33. Scale bar, 100 µm. HE, hematoxylin–eosin. **d** Representative images of immunohistochemical staining of consecutive sections of Tumor 22 showing an intermingled expression pattern of ASCL1 and NEUROD1. Scale bar, 100 µm. **e** Representative images of multiplex immunofluorescence staining of ASCL1 and NEUROD1 in Tumor 22. Scale bar, 50 µm. **f** The proportions of ASCL1- and/or NEUROD1-positive cells by multiplex immunofluorescence staining of six SCLC-A tumors co-expressing NEUROD1 and of four SCLC-N tumors co-expressing ASCL1. Dual indicates that both ASCL1 and NEUROD1 are positive in a single tumor cell.

First, we characterized p53 and Rb expression patterns (Supplementary Fig. 1a) in a total of 151 surgically treated human SCLC tumors and found that 95.2% and 90.7% of the tumors had inactivated p53 (Supplementary Fig. 1b) and inactivated Rb (Supplementary Fig. 1c) patterns, respectively. The dual-inactivated status was most frequently observed (*N* = 131, 86.7%), followed by the p53-inactivated and Rb-wild-type (WT) status (*N* = 12, 7.9%) and the p53-WT and Rb-inactivated status (*N* = 5, 3.3%). We then evaluated the protein expression patterns of the four lineage-specific TFs (ASCL1, NEUROD1, POU2F3, and YAP1) in these tumors (Fig. 1a and Supplementary Fig. 1d). The unavailability of appropriate antibodies targeting ATOH1 for immunohistochemistry (IHC) analysis precluded the assessment of ATOH1 in this study. The patient and tumor characteristics are shown in Supplementary Table 1; data interpretation should take into account the exclusive collection of surgically treated tumors in this study to ensure sufficient quality and quantity for pathological evaluation. Despite some TF overlaps in individual tumor samples, we were able to classify the tumors as SCLC-A (*N* = 90, 59.6%), SCLC-N (*N* = 14, 9.2%), SCLC-P (*N* = 36, 23.8%), and SCLC-Y (*N* = 10, 6.6%), except for one tumor (0.6%) that was quadruple-negative (SCLC-QN; Fig. 1a, b). Along with the frequent co-expression of POU2F3 and YAP1 (both showing H-scores >10), co-expression of ASCL1 and NEUROD1 was observed in 23 samples (15.2%). Detailed evaluation of these 23 samples co-expressing ASCL1 and NEUROD1 revealed that, for one sample, there was a clear boundary between the components positive for each of ASCL1 or NEUROD1, with no transitional alterations (Tumor 33; Fig. 1c). The remaining 22 samples showed an intermingled expression pattern of ASCL1 and NEUROD1 in the same tumor areas (Fig. 1d). To further explore the expression status at the cellular resolution in these cases with mixed expression, we performed double-staining immunofluorescence of ASCL1 and NEUROD1 (Fig. 1e and Supplementary Fig. 1e). This analysis was successful in 10 of 22 samples, all of which demonstrated a high degree of mutual exclusivity in the cellular expression of ASCL1 and NEUROD1 (Fig. 1f), with the median proportions of dual-positive cells being 4.5% (range, 2.1%–6.8%) for SCLC-A tumors co-expressing NEUROD1 and 5.0% (range, 3.7%–5.7%) for SCLC-N tumors co-expressing ASCL1. Together, these results suggest that lineage-specific TFs are largely expressed in clinical SCLC specimens in a mutually exclusive manner at the tumor level, whereas this principle applies more at the cellular level for ASCL1 and NEUROD1 expression.

### Mutual repression between lineage-specific TFs in human SCLC cell lines

**Fig. 2:**
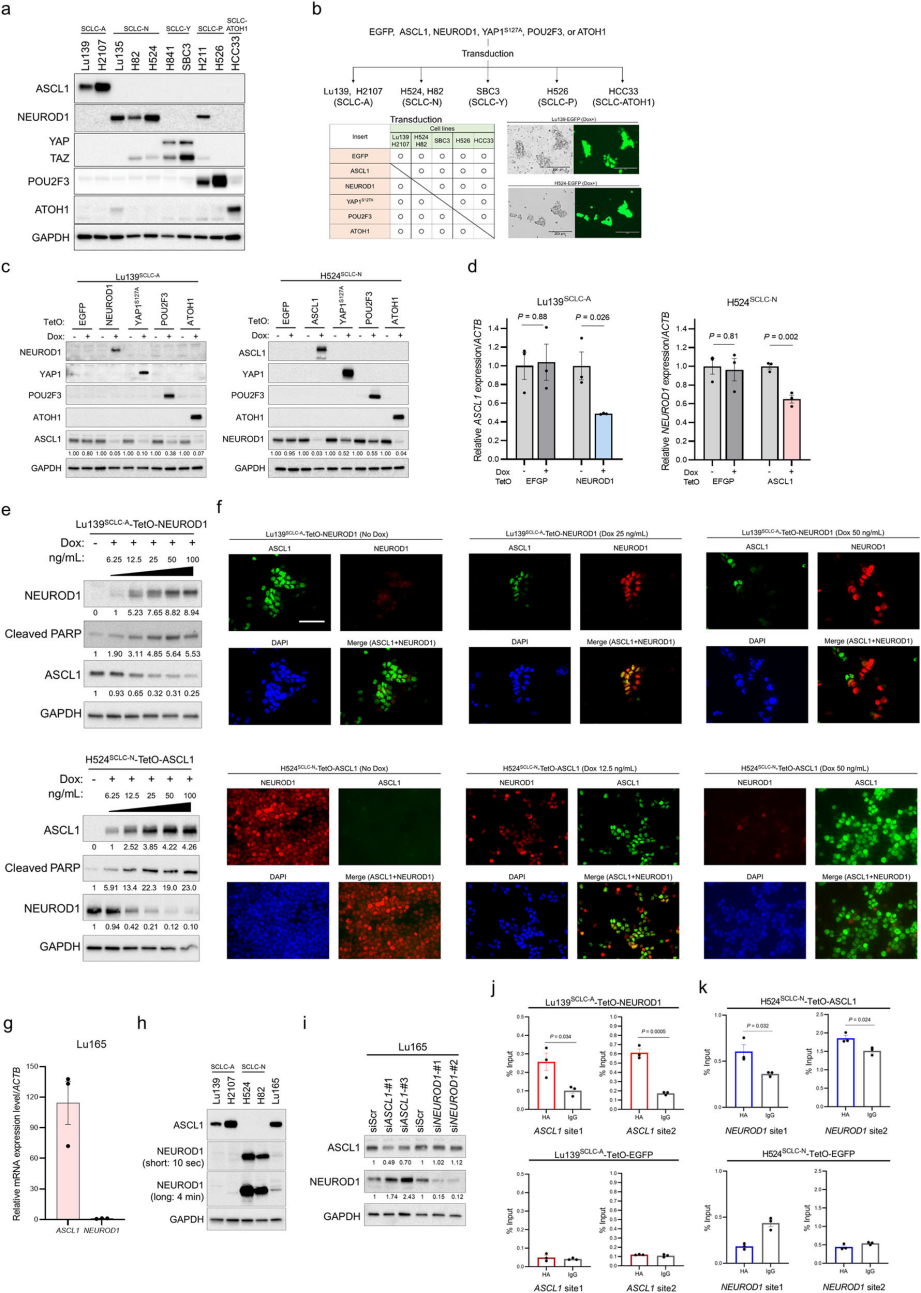
Mutual repression of lineage-specific transcription factors (TFs) in the human SCLC cell line co-expression models. **a** Immunoblots showing the expression profiles of the five intrinsic lineage-specific TFs in human SCLC cell lines. **b** Schema of the establishment of the co-expression models of lineage-specific TFs in SCLC cell lines. ASCL1-dominant (SCLC-A; Lu139 and H2107), NEUROD1-dominant (SCLC-N; H524 and H82), YAP1-dominant (SCLC-Y; SBC3), POU2F3-dominant (SCLC-P; H526), and ATOH1-dominant (HCC33) cell lines were used. Enhanced green fluorescent protein (EGFP)-expressing cells were used as controls. **c** Immunoblots showing the expression of lineage-specific TFs in Lu139^SCLC-A^ (left) and H524^SCLC-N^ (right) derivatives before and 5 days after doxycycline (Dox)-mediated induction of NEUROD1, YAP1^S127A^, POU2F3, or ATOH1 for Lu139^SCLC-A^, and of ASCL1, YAP1^S127A^, POU2F3, or ATOH1 for H524^SCLC-N^. The numbers below the ASCL1 and NEUROD1 blots indicate the values of the bands relative to the corresponding non-doxycycline control values after normalization against GAPDH. **d** Expression levels of endogenous *ASCL1* transcripts after NEUROD1 co-expression in Lu139^SCLC-A^ cells (left) and of endogenous *NEUROD1* transcripts after ASCL1 co-expression in H524^SCLC-N^ cells (right), relative to non-doxycycline controls after normalization against *ACTB* expression. The corresponding EGFP-expressing cells were used as controls. Cells were harvested after treatment with doxycycline for 72 hours. **e** Immunoblots showing changes in endogenous ASCL1 expression in Lu139^SCLC-A^ cells (top) and endogenous NEUROD1 expression in H524^SCLC-N^ cells (bottom) after incrementally increased co-expression of NEUROD1 and ASCL1, respectively. Cells were treated with doxycycline for 5 days. The numbers below the NEUROD1, ASCL1, and cleaved PARP blots indicate the values of the bands relative to the corresponding non-doxycycline control values after normalization against GAPDH. **f** Representative images of multiplex immunofluorescence staining of ASCL1 and NEUROD1 in Lu139^SCLC-A^-TetO-NEUROD1 cells (top) and H524^4SCLC-N^-TetO-ASCL1 cells (bottom) after treatment with different concentrations of doxycycline for 72 hours. Scale bar, 50 µm. **g** The expression level of *ASCL1* mRNA relative to *NEUROD1* mRNA in Lu165 cells. **h** Immunoblots showing the protein expression of ASCL1 and NEUROD1 in Lu165 cells. NEUROD1 is presented with short exposure (10 seconds) and long exposure (4 minutes) images. Lu139^SCLC-A^ cells and H2107^SCLC-A^ cells were used as positive controls for ASCL1 expression, while H524^SCLC-N^ cells and H82^SCLC-N^ cells were used as positive controls for NEUROD1 expression. **i** Immunoblots showing the expression of ASCL1 and NEUROD1 under *ASCL1* or *NEUROD1* knockdown in Lu165 cells. Cells were harvested 5 days after transfection of indicated siRNAs. The numbers below the ASCL1 and NEUROD1 blots indicate the values of the bands relative to the corresponding scrambled siRNA (siScr) control values after normalization against GAPDH. **j** CUT&RUN assay and quantitative PCR for binding sites of exogenous NEUROD1 on the *ASCL1* loci in Lu139^SCLC-A^-TetO-NEUROD1 cells (top). Lu139^SCLC-A^-TetO-EGFP-expressing cells were used as a control (bottom). The anti-HA-tag antibody was used to detect exogenous NEUROD1 and EGFP with C-terminal HA-tags, and the rabbit IgG isotype control was used as a negative control. The amount of obtained DNA in each sample is represented as a signal relative to the total amount of input chromatin. **k** CUT&RUN assay and quantitative PCR for binding sites of exogenous ASCL1 on the *NEUROD1* loci in H524^SCLC-N^-TetO-ASCL1 cells (top). H524^SCLC-N^-TetO-EGFP cells were used as a control (bottom). The anti-HA-tag antibody was used to detect exogenous ASCL1 and EGFP with C-terminal HA-tags, and the rabbit IgG isotype control was used as a negative control. The amount of obtained DNA in each sample is represented as a signal relative to the total amount of input chromatin. For CUT&RUN assays, cells were treated with doxycycline for 72 hours (**j**, **k**). Each Immunoblot image is representative of at least two independent procedures (**a**, **c**, **e**, **h**, **i**). Error bars indicate ± the standard error of the mean (SEM) from *N* = 3 independent experiments (**d**, **g**, **j**, **k**). Student’s t-test was used for two-group comparisons (**d**, **j**, **k**).

Immunoblot analysis revealed that the five lineage-specific TFs (ASCL1, NEUROD1, YAP1, POU2F3, and ATOH1) are more obviously expressed in a mutually exclusive manner in human SCLC cell lines than in clinical SCLC specimens, with NCI-H211 (H211) cells as an exception, showing clear co-expression of NEUROD1 and POU2F3 (Fig. 2a). On the basis of these data, we established inducible TF co-expression models in SCLC cell lines of all the combinations (Fig. 2b). Specifically, Lu139 cells (SCLC-A), NCI-H524 (H524) cells (SCLC-N), SBC3 cells (SCLC-Y), NCI-H526 (H526) cells (SCLC-P), and HCC33 cells (SCLC-ATOH1) were used for this Tet-On expression system in which the five TFs could all be co-expressed under the tight control of the doxycycline-inducible TetO promoter. For a YAP1-expression model, YAP1^S127A^ was used to ensure the continuous transcriptional activation of YAP in the nucleus^22^. Enhanced green fluorescent protein (EGFP)-expressing cells were used as corresponding controls (Fig. 2b). Notably, co-expression of NEUROD1, YAP1^S127A^, or ATOH1 in Lu139^SCLC-A^ cells strongly suppressed the endogenous expression of ASCL1 protein (Fig. 2c). In H524^SCLC-N^ cells, overexpression of ASCL1 or ATOH1 similarly suppressed the endogenous NEUROD1 expression. Co-expression of YAP1^S127A^ suppressed endogenous POU2F3 expression in H526^SCLC-P^ cells, while ASCL1 and NEUROD1 suppressed endogenous ATOH1 expression in HCC33^SCLC-ATOH1^ cells (Supplementary Fig. 2a). These findings were validated in a second cell line for the SCLC-A and SCLC-N models, using NCI-H2107 (H2107) and NCI-H82 (H82) cells, respectively (Supplementary Fig. 2b). Mutual suppression between *ASCL1* and *NEUROD1* was confirmed at the transcriptomic level (Fig. 2d). Furthermore, the suppression of ASCL1 or NEUROD1 occurred in a manner dependent on the dose of doxycycline, with an increase in the levels of cleaved PARP (Fig. 2e), one of the markers of apoptosis. Double immunofluorescence staining of ASCL1 and NEUROD1 revealed that ASCL1 was weakly co-expressed with NEUROD1 in some Lu139^SCLC-A^-TetO-NEUROD1 cells at a low level (25 ng/mL) of doxycycline, while ASCL1 was expressed exclusively in cells lacking NEUROD1 expression at a higher level (50 ng/mL) of doxycycline (Fig. 2f). In H524^SCLC-N^-TetO-ASCL1 cells, NEUROD1 was almost exclusively expressed in cells lacking ASCL1 expression even at a low level (12.5 ng/mL) of doxycycline (Fig. 2f). These results suggest that, although weak co-expression of NEUROD1 in SCLC-A cells can allow ASCL1 co-expression in some cells, co-expression of ASCL1 in SCLC-N cells does not allow such a gradual effect and causes TF switching. Furthermore, these results indicate that repressed, but still detectable, expression of ASCL1 in Lu139^SCLC-A^-TetO-NEUROD1 cells and NEUROD1 in H524^SCLC-N^-TetO-ASCL1 cells at higher doses of doxycycline treatment was derived from a minor population of cells lacking induction of the other TFs, which was caused by the heterogeneity of cells based on the polyclonal experimental design. As Lu165 cells co-express ASCL1 and NEUROD1^23^, we next quantified the expression levels of both TFs in this cell line and revealed that the expression of ASCL1 was much greater than that of NEUROD1 at both the mRNA (Fig. 2g) and protein (Fig. 2h) levels. Furthermore, knockdown of *ASCL1* in Lu165 cells resulted in increased NEUROD1 expression, whereas knockdown of *NEUROD1* did not enhance ASCL1 expression (Fig. 2i). Together, these results suggest that most lineage-specific TFs, if not all, exert mutually repressive functions, and that some combinations of TFs, such as ASCL1 and NEUROD1, cannot be co-expressed above certain thresholds in SCLC cells.

Considering the magnitude of the discrepancy in the repression of endogenous ASCL1 and NEUROD1 at the protein (by up to 97%; Fig. 2c) and mRNA (by up to 52%; Fig. 2d) levels, we next explored whether post-translational modifications were involved in this repression. To this end, we treated Lu139^SCLC-A^-TetO-NEUROD1 cells and H524^SCLC-N^-TetO-ASCL1 cells with a proteasome inhibitor, MG132. However, neither ASCL1 nor NEUROD1 expression was restored, in contrast to the increased expression of Cyclin B1, a known target of the proteasome^24^ (Supplementary Fig. 2c). These findings suggest that the ubiquitin-proteasome system is not involved in the regulation of ASCL1 and NEUROD1 in our model. To gain mechanistic insights into the mutual repression between ASCL1 and NEUROD1, we next performed Cleavage Under Targets and Release Using Nuclease (CUT&RUN)-quantitative PCR (Supplementary Fig. 2d, e) and found that NEUROD1 bound to the binding motif in the *ASCL1* gene (Fig. 2j) and vice versa (Fig. 2k). These data suggest that ASCL1 and NEUROD1 may transcriptionally repress each other by binding to the loci of each other’s genes.

### Co-expression of lineage-specific TFs induces apoptosis in SCLC cells

**Fig. 3:**
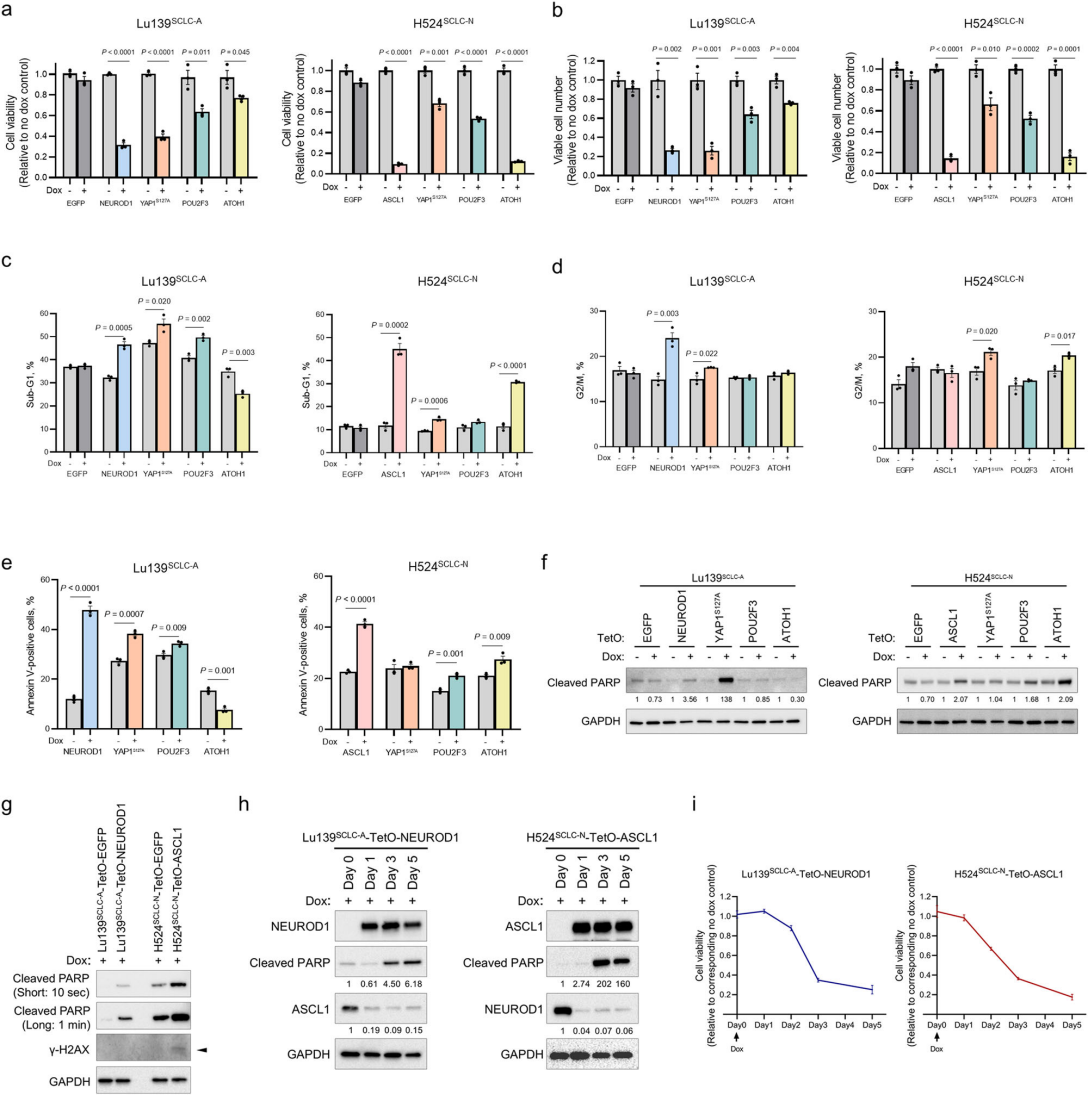
Phenotypic consequences after the co-expression of lineage-specific transcription factors (TFs) in SCLC cell lines. **a** Quantification of cell viability relative to corresponding non-doxycycline control cells in Lu139^SCLC-A^ (left) and H524^SCLC-N^ (right) derivatives 5 days after co-expression of NEUROD1, YAP1^S127A^, POU2F3, or ATOH1 with ASCL1 in Lu139^SCLC-A^ cells, and of ASCL1, YAP1^S127A^, POU2F3, or ATOH1 with NEUROD1 in H524^SCLC-N^ cells. Enhanced green fluorescent protein (EGFP) cells were used as controls. Cell viability was assessed using alamarBlue Cell Viability Regent. **b** Viable cell numbers relative to corresponding non-doxycycline control cells in Lu139^SCLC-A^ (left) and H524^SCLC-N^ (right) derivatives 5 days after co-expression of NEUROD1, YAP1^S127A^, POU2F3, or ATOH1 with ASCL1 in Lu139^SCLC-A^ cells and of ASCL1, YAP1^S127A^, POU2F3, or ATOH1 with NEUROD1 in H524^SCLC-N^ cells. EGFP cells were used as controls. Viable cell numbers were counted using Trypan Blue. **c** Proportions of cells in the sub-G_1_ phase in Lu139^SCLC-A^ (left) and H524^SCLC-N^ (right) derivatives with or without co-expression of NEUROD1, YAP1^S127A^, POU2F3, or ATOH1 with ASCL1 in Lu139^SCLC-A^ cells and of ASCL1, YAP1^S127A^, POU2F3, or ATOH1 with NEUROD1 in H524^SCLC-N^ cells. EGFP cells were used as controls. Cells were treated with doxycycline for 5 days. **d** Proportions of cells in the G_2_/M phase in Lu139^SCLC-A^ (left) and H524^SCLC-N^ (right) derivatives with or without co-expression of NEUROD1, YAP1^S127A^, POU2F3, or ATOH1 with ASCL1 in Lu139^SCLC-A^ cells and of ASCL1, YAP1^S127A^, POU2F3, or ATOH1 with NEUROD1 in H524^SCLC-N^ cells. EGFP cells were used as controls. Cells were treated with doxycycline for 5 days. **e** Proportions of annexin V-positive cells in Lu139^SCLC-A^ (left) and H524^SCLC-N^ (right) derivatives with or without co-expression of NEUROD1, YAP1^S127A^, POU2F3, or ATOH1 with ASCL1 in Lu139^SCLC-A^ cells and of ASCL1, YAP1^S127A^, POU2F3, or ATOH1 with NEUROD1 in H524^SCLC-N^ cells. Cells were treated with doxycycline for 5 days. **f** Immunoblots showing the expression of cleaved PARP in Lu139^SCLC-A^ (left) and H524^SCLC-N^ (right) derivatives with or without co-expression of NEUROD1, YAP1^S127A^, POU2F3, or ATOH1 with ASCL1 in Lu139^SCLC-A^ cells and of ASCL1, YAP1^S127A^, POU2F3, or ATOH1 with NEUROD1 in H524^SCLC-N^ cells. EGFP cells were used as controls. Cells were treated with doxycycline for 5 days. The numbers below the cleaved PARP blots indicate the values of bands relative to the corresponding non-doxycycline control values after normalization against GAPDH. **g** Immunoblots showing the expression of cleaved PARP and γ-H2AX in Lu139^SCLC-A^-TetO-NEUROD1 cells and H524^SCLC-N^-TetO-ASCL1 cells 5 days after treatment with doxycycline. Cleaved PARP is presented with short exposure (10 seconds) and long exposure (1 min) images. Corresponding EGFP cells were used as controls. The arrow indicates the γ-H2AX band. **h** Time course expression of exogenous NEUROD1, endogenous ASCL1, and cleaved PARP in Lu139^SCLC-A^-TetO-NEUROD1 cells after the initiation of doxycycline treatment (left) and that of exogenous ASCL1, endogenous NEUROD1, and cleaved PARP in H524^SCLC-N^-TetO-ASCL1 cells after the initiation of doxycycline treatment (right). The numbers below the cleaved PARP blots and endogenous ASCL1 or NEUROD1 indicate the values of bands relative to the corresponding Day 0 control values after normalization against GAPDH. **i** Time course quantification of cell viability relative to the corresponding non-doxycycline control cells in Lu139^SCLC-A^-TetO-NEUROD1 cells (left) and H524^SCLC-N^-TetO-ASCL1 cells (right) after the initiation of doxycycline treatment. Cell viability was assessed using alamarBlue Cell Viability Regent. Each Immunoblot image is representative of at least two independent procedures (**f**–**h**). Error bars indicate ± the standard error of the mean (SEM) from *N* = 3 independent experiments (**a**–**e**, **i**). Student’s *t* test was used for two-group comparisons (**a**–**e**).

We next assessed the biological effects of co-expression of these lineage-specific TFs. In Lu139^SCLC-A^ and H524^SCLC-N^ derivatives, co-expression of any TF other than EGFP significantly reduced cell viability and viable cell numbers, with the most striking effects being observed with NEUROD1 or YAP1^S127A^ co-expression with ASCL1 in Lu139^SCLC-A^ cells and with ASCL1 or ATOH1 co-expression with NEUROD1 in H524^SCLC-N^ cells (Fig. 3a, b). Although the inhibitory effects of YAP1^S127A^ co-expression in SCLC-A cells and of ASCL1 or YAP1^S127A^ co-expression in SCLC-N cells were validated by a cell viability assay in H2107^SCLC-A^ and H82^SCLC-N^ cells, respectively, co-expression of NEUROD1 with ASCL1 in H2107^SCLC-A^ cells did not result in a significant decrease in cell viability (Supplementary Fig. 3a). In SBC3^SCLC-Y^, H526^SCLC-P^, and HCC33^SCLC-ATOH1^ cells, the co-expression of lineage-specific TFs had variable effects on cell viability (Supplementary Fig. 3b). However, the EGFP controls also showed decreased cell viability in these three cell lines for an unknown reason. This excluded the possibility of a toxic effect induced by doxycycline itself (Supplementary Fig. 3c, d). Precise evaluation of cell viability after TF co-expression was, therefore, not possible in these three cell lines. A cell cycle assay revealed that the proportions of cells in sub-G_1_ phase were significantly increased on co-expression of NEUROD1, YAP1^S127A^, or POU2F3 with ASCL1 in Lu139^SCLC-A^ cells, and on co-expression of ASCL1, YAP1^S127A^, or ATOH1 with NEUROD1 in H524^SCLC-N^ cells (Fig. 3c and Supplementary Fig. 3e), indicative of increased apoptosis in these cells^25^. After removal of the sub-G_1_ phase populations from Lu139^SCLC-A^ and H524^SCLC-N^ derivatives, co-expression of NEUROD1 or YAP1^S127A^ with ASCL1 in Lu139^SCLC-A^ cells, and of YAP1^S127A^ or ATOH1 with NEUROD1 in H524^SCLC-N^ cells, led to significant increases in the G_2_/M populations (Fig. 3d and Supplementary Fig. 3e, f). Accordingly, the co-expression of NEUROD1 or ASCL1 in Lu139^SCLC-A^ or H524^SCLC-N^ cells, respectively, also resulted in marked increases in apoptotic cells in the annexin V-FITC apoptosis assay (Fig. 3e and Supplementary Fig. 3g). This analysis also showed significant increases in apoptosis after co-expression of YAP1^S127A^ or POU2F3 with ASCL1 in Lu139^SCLC-A^ cells, and after co-expression of POU2F3 or ATOH1 with NEUROD1 in H524^SCLC-N^ cells (Fig. 3e and Supplementary Fig. 3g). This increased apoptosis was validated by immunoblotting for cleaved PARP in Lu139^SCLC-A^ cells co-expressing NEUROD1 or YAP1^S127A^ with ASCL1 and in H524^SCLC-N^ cells co-expressing ASCL1, POU2F3, or ATOH1 with NEUROD1 (Figs. 2e and 3f).

As ASCL1 and NEUROD1 are the most prevalent lineage factors expressed in the majority of SCLC cases^8,12^, and as their co-expression exerted strong detrimental effects in our model, we subsequently focused on investigating the biological consequences of ASCL1 and NEUROD1 co-expression. Loss of Rb has been shown to result in the absence or reduction of Cyclin D1 expression, a phenomenon attributed to elevated p16 protein levels which is associated with Rb inactivation^26^. Consistent with this, no Cyclin D1 expression was detected in Rb-inactivated SCLC cell lines (Lu139^SCLC-A^, H2107^SCLC-A^, H82^SCLC-N^, H526^SCLC-P^, and HCC33^SCLC-ATOH1^), with the exception of H524^SCLC-N^ (Supplementary Fig. 3h). Furthermore, Cyclin D1 expression was not induced in Lu139^SCLC-A^-TetO-NEUROD1 cells and was downregulated in H524^SCLC-N^-TetO-ASCL1 cells (Supplementary Fig. 3h). However, these changes in Cyclin D1 expression were not associated with the alterations in cell cycle profiles (Supplementary Fig. 3f). To investigate the involvement of DNA damage in apoptotic cell death, we evaluated γ-H2AX expression, an established marker of DNA damage. A modest increase in γ-H2AX was observed in H524^SCLC-N^-TetO-ASCL1 cells, but not in Lu139^SCLC-A^-TetO-NEUROD1 cells (Fig. 3g). Lastly, we monitored the repression of endogenous subtype-defining TFs, loss of cell viability, and apoptosis over time. The downregulation of ASCL1 in Lu139^SCLC-A^-TetO-NEUROD1 cells and NEUROD1 in H524^SCLC-N^-TetO-ASCL1 cells was evident as early as on day 1 following doxycycline treatment (Fig. 3h), preceding both the loss of cell viability (Fig. 3i) and the onset of apoptotic cell death (Fig. 3h). These findings suggest that the downregulation of endogenous ASCL1 and NEUROD1 is not a secondary consequence of reduced cell viability or apoptosis. Collectively, our data demonstrate that, among all the combinations of the lineage-specific TFs, the co-expression of ASCL1 and NEUROD1 in SCLC cells is notably detrimental by inducing apoptosis following the downregulation of endogenous lineage-specific TFs. A point of caution is that this may be more universal in SCLC-N cell lines than in SCLC-A cell lines.

### ASCL1 and NEUROD1 mutually suppress gene expression programs driven by their counterpart in SCLC cell lines

**Fig. 4:**
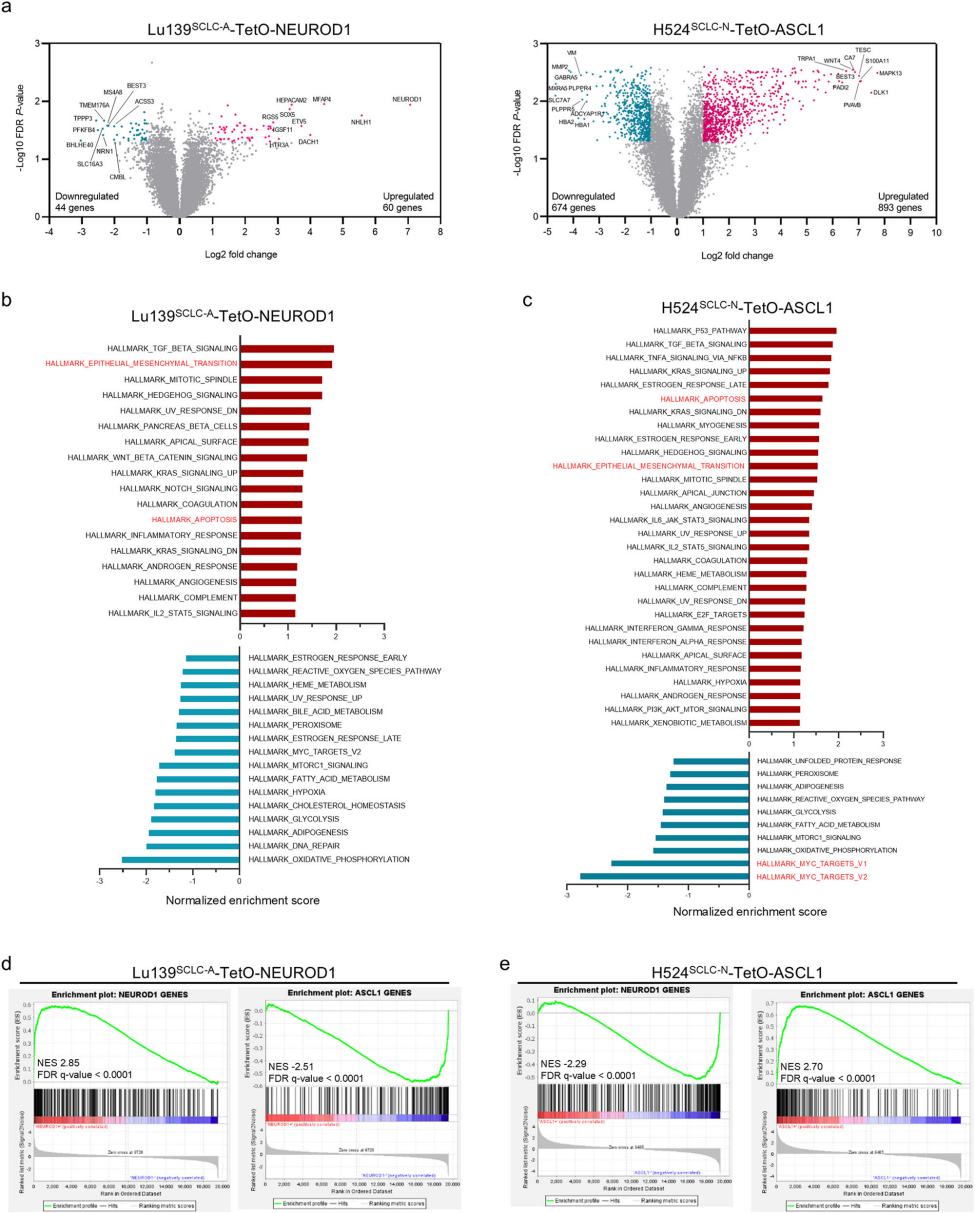
Gene expression reprogramming after co-expression of ASCL1 and NEUROD1 in SCLC cell lines. **a** The distributions of differentially expressed genes in Lu139^SCLC-A^-TetO-NEUROD1 cells (left) and H524^SCLC-N^-TetO-ASCL1 cells (right) compared with corresponding non-doxycycline treatment controls. Cells were treated with doxycycline for 72 hours. Significantly differentially expressed genes are shown in blue (downregulated) or red (upregulated). Samples were analyzed in triplicate. **b**, **c** Upregulated (red) and downregulated (blue) hallmark gene sets analyzed by gene set enrichment analysis (GSEA) in Lu139^SCLC-A^-TetO-NEUROD1 cells (**b**) and H524^SCLC-N^-TetO-ASCL1 cells (**c**). The bars are arranged based on the normalized enrichment scores. **d**, **e** GSEA plots of the established NEUROD1 target genes and ASCL1 target genes in Lu139^SCLC-A^-TetO-NEUROD1 cells (**d**) and in H524^SCLC-N^-TetO-ASCL1 cells (**e**), compared with the corresponding non-doxycycline treatment controls. NES, normalized enrichment score.

We sought to determine whether the observed mutual repression and phenotypic effects were coordinated with changes in gene expression. Gene expression analyses of Lu139^SCLC-A^-TetO-NEUROD1 cells and H524^SCLC-N^-TetO-ASCL1 cells with or without doxycycline treatment revealed distinct transcriptional profiles that included 104 and 1567 differentially expressed genes, respectively (Fig. 4a). Gene set enrichment analysis (GSEA) revealed that, in Lu139^SCLC-A^-TetO-NEUROD1 cells with doxycycline treatment, gene sets associated with TGF-β signaling, the epithelial–mesenchymal transition (EMT), and apoptosis were upregulated, whereas gene sets such as oxidative phosphorylation and DNA repair were downregulated (Fig. 4b and Supplementary Fig. 4a). In H524^SCLC-N^-TetO-ASCL1 cells with doxycycline treatment, gene sets associated with the p53 pathway, TGF-β signaling, apoptosis, and EMT were upregulated (Fig. 4c and Supplementary Fig. 4a). Immunoblot analysis of EMT-associated molecules confirmed upregulation of N-cadherin and Slug and downregulation of E-cadherin in Lu139^SCLC-A^-TetO-NEUROD1 cells and upregulation of Snail and Slug in H524^SCLC-N^-TetO-ASCL1 cells (Supplementary Fig. 4b).

MYC is crucial in driving SCLC-N^27^ and maintaining NEUROD1 expression^15^. As such, the H524^SCLC-N^ cell line is a high-MYC cell line^28^. Interestingly, MYC target genes were markedly downregulated in H524^SCLC-N^-TetO-ASCL1 cells (Fig. 4c and Supplementary Fig. 4c). Moreover, MYC expression itself was suppressed at both the transcriptional and protein levels (Supplementary Fig. 4d, e). This was validated by Enrichr analysis showing that *MYC*, along with its heterodimerizing partner *MAX*, were the top downregulated genes in H524^SCLC-N^-TetO-ASCL1 cells (Supplementary Fig. 4f).

Of particular note, GSEA using established human ASCL1 or NEUROD1 target genes^29^ in Lu139^SCLC-A^-TetO-NEUROD1 cells revealed significant upregulation of target genes of the exogenously expressed NEUROD1 and a significant depletion of target genes of the endogenous ASCL1 (Fig. 4d), which was mirrored in H524^SCLC-N^-TetO-ASCL1 cells (Fig. 4e). These data demonstrate that ASCL1 and NEUROD1 possess the capacity to reprogram the cell differentiation program. We next asked whether ASCL1 and NEUROD1 could physically interact and modulate each other’s transcriptional activity, similar to the interaction reported between MYC and MYC-interacting zinc finger protein 1^30^. However, a proximity ligation assay performed in Lu139^SCLC-A^-TetO-NEUROD1 cells provided no evidence of direct interaction between these TFs (Supplementary Fig. 4g).

### Co-expression of ASCL1 and NEUROD1 changes the chromatin accessibility and rewires the cell differentiation program

**Fig. 5:**
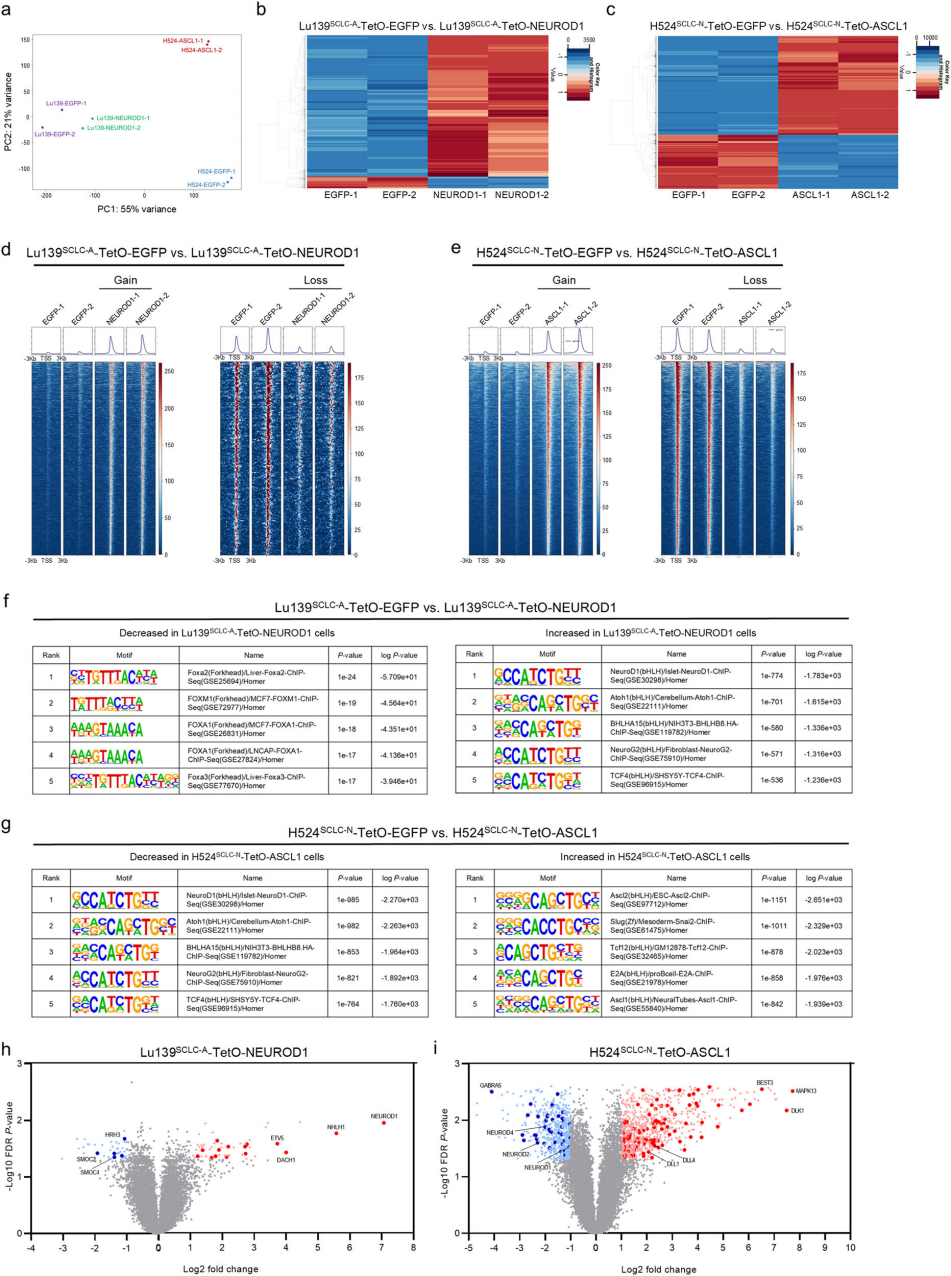
Co-expression of ASCL1 and NEUROD1 rewires the chromatin accessibility landscape in ASCL1- or NEUROD1-driven SCLC cells. **a** Principal component analysis of all the chromatin accessibility peaks from ATAC-seq (assay for transposase-accessible chromatin with sequencing) data of Lu139^SCLC-A^-TetO-EGFP, Lu139^SCLC-A^-TetO-NEUROD1, H524^SCLC-N^-TetO-EGFP, and H524^SCLC-N^-TetO-ASCL1 cells. For ATAC-seq, cells were treated with doxycycline for 72 hours in duplicate. **b** Heatmap showing the significantly differential peak regions between Lu139^SCLC-A^ cells co-expressing ASCL1 with EGFP and NEUROD1. **c** Heatmap showing the significantly differential peak regions between H524^SCLC-N^ cells co-expressing NEUROD1 with EGFP and ASCL1. **d**, **e** Signal aggregation plots and signal heatmaps showing the significantly differential DNA accessible regions (gain and loss) in Lu139^SCLC-A^-TetO-NEUROD1 cells compared with those in Lu139^SCLC-A^-TetO-EGFP cells (**d**), and in H524^SCLC-N^-TetO-ASCL1 cells compared with those in H524^SCLC-N^-TetO-EGFP cells (**e**). **f**, **g** HOMER motif enrichment analysis of the ATAC-seq data showing the top five decreased (left) and increased (right) regulatory motifs in Lu139^SCLC-A^-TetO-NEUROD1 cells compared with those in Lu139^SCLC-A^-TetO-EGFP cells (**f**), and in H524^SCLC-N^-TetO-ASCL1 cells compared with those in H524^SCLC-N^-TetO-EGFP cells (**g**). **h**, **i** Volcano plots showing the integration of the differential gene expression analysis from the microarray and ATAC-seq data. Genes near the differential peaks detected by ATAC-seq when comparing Lu139^SCLC-A^-TetO-NEUROD1 and Lu139^SCLC-A^-TetO-EGFP cells or H524^SCLC-N^-TetO-ASCL1 and H524^SCLC-N^-TetO-EGFP cells were nominated with the threshold of log_2_(fold change) >1. Among these genes, the ASCL1- and NEUROD1-related genes were selected and are highlighted in dark red or dark blue, respectively, on the volcano plots if they showed significant differences in gene expression, based on the criteria of log_2_(fold change) >1 and FDR *P* value < 0.05.

As ASCL1 and NEUROD1 are pioneer factors^31–34^, possessing the unique ability to bind DNA target sites within closed chromatin and initiate chromatin reorganization and opening^35^, we hypothesized that the co-expression of ASCL1 and NEUROD1 could directly rewire the chromatin accessibility in our model. Assessment of the chromatin accessibility landscape by bulk assay for transposase-accessible chromatin with sequencing (ATAC-seq), comparing Lu139^SCLC-A^-TetO-NEUROD1 with Lu139^SCLC-A^-TetO-EGFP control cells, and H524^SCLC-N^-TetO-ASCL1 with H524^SCLC-N^-TetO-EGFP control cells, revealed a more pronounced effect on H524^SCLC-N^-TetO-ASCL1 cells than on Lu139^SCLC-A^-TetO-NEUROD1 cells compared with their respective controls, as shown by principal component analysis (Fig. 5a). This is consistent with the results of the gene expression analysis (Fig. 4a). Specifically, co-expression of NEUROD1 in Lu139^SCLC-A^ cells caused an overall increase in chromatin accessibility (2425 peaks of chromatin accessibility gained, 200 lost; Fig. 5b), while co-expression of ASCL1 in H524^SCLC-N^ cells also caused an overall increase in chromatin accessibility (6463 peaks of chromatin accessibility gained, 3528 lost; Fig. 5c). Signal aggregation plots and a signal heatmap of the differential accessible DNA regions revealed clear differences after the co-expression of NEUROD1 and ASCL1 in Lu139^SCLC-A^ (Fig. 5d) and H524^SCLC-N^ (Fig. 5e) cells. HOMER motif enrichment analysis showed that NEUROD1 co-expression with endogenous ASCL1 in Lu139^SCLC-A^ cells led to significantly increased accessibility at the NEUROD1 motif, which was highest ranked (Fig. 5f); the FOXA2 motif was the top significantly decreased regulatory motif (Fig. 5f). In H524^SCLC-N^ cells, ASCL1 co-expression with endogenous NEUROD1 resulted in increased accessibility at the ASCL2 (ranked 1) and ASCL1 (ranked 5) motifs (Fig. 5g); notably, the NEUROD1 motif was the most significantly decreased regulatory motif (Fig. 5g). Compared with EGFP control cells, Lu139^SCLC-A^-TetO-NEUROD1 cells had decreased chromatin accessibility at the endogenous *ASCL1* loci, although this was not statistically significant (Supplementary Fig. 5a), and H524^SCLC-N^-TetO-ASCL1 cells showed significantly decreased chromatin accessibility at the endogenous *NEUROD1* loci (Supplementary Fig. 5b). These findings indicate that mutual repression between ASCL1 and NEUROD1 could be partly due to the loss of chromatin accessibility at these gene loci. Next, we generated volcano plots integrating the chromatin accessibility data and the differential expression analysis of the gene expression data. In Lu139^SCLC-A^-TetO-NEUROD1 cells, 17 of the 792 NEUROD1-related genes were both significantly upregulated and gained chromatin accessibility, while five of the 853 ASCL1-related genes were both significantly downregulated and showed decreased chromatin accessibility (Fig. 5h). In H524^SCLC-N^-TetO-ASCL1 cells, 96 of the 833 ASCL1-related genes were both significantly upregulated and gained accessibility, while 40 of the 792 NEUROD1-related genes were both significantly downregulated and lost accessibility (Fig. 5i). Together, these data suggest that both ASCL1 and NEUROD1 can rewire the chromatin accessibility when co-expressed in cells driven by their counterpart. Co-expression of ASCL1 in NEUROD1-driven cells rewired chromatin with greater efficiency, giving a more pronounced impact on gene expression reprogramming and potentially more significant biological consequences.

### Derepression of the endogenous TFs does not fully rescue the decreased cell viability caused by co-expression of alternative TFs

**Fig. 6:**
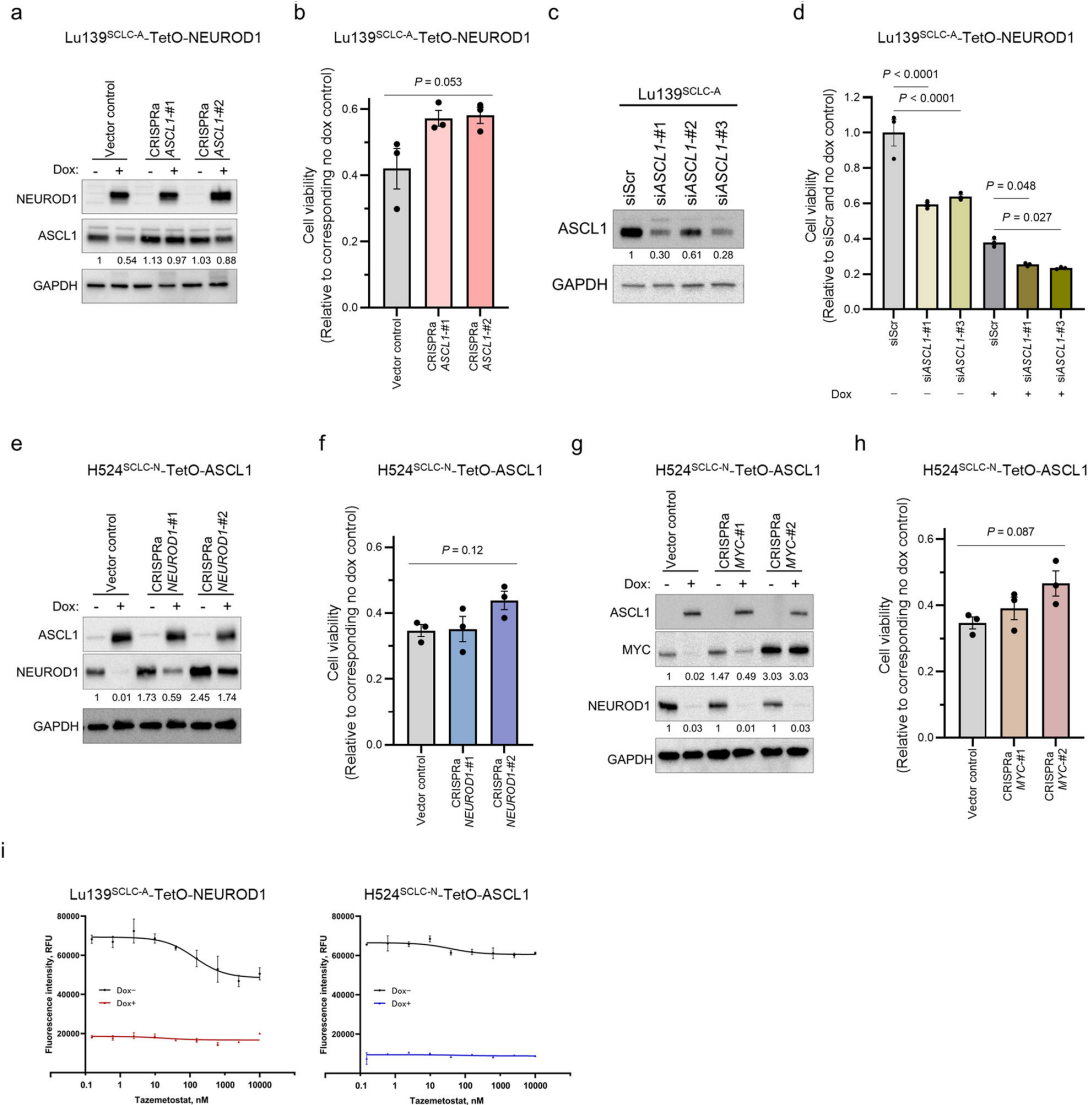
Assessment of cell viability rescue by CRISPRa-mediated derepression of the suppressed transcription factor (TF) in ASCL1 and NEUROD1 co-expression models. **a** Immunoblots showing the expression of NEUROD1 and ASCL1 in Lu139^SCLC-A^-TetO-NEUROD1 cells with (for 5 days) or without doxycycline treatment under activation of endogenous *ASCL1* by the CRISPR activation (CRISPRa) system. The numbers below the ASCL1 blots indicate the values of the bands relative to the non-doxycycline-treated CRISPRa vector control value after normalization against GAPDH. **b** Quantification of cell viability 5 days after treatment with doxycycline relative to corresponding non-doxycycline control cells in Lu139^SCLC-A^ cells co-expressing NEUROD1 with ASCL1 after the CRISPRa-mediated activation of *ASCL1*. **c** Immunoblot showing the expression of ASCL1 in Lu139^SCLC-A^ cells under *ASCL1* knockdown with three siRNAs (5 days after transfection). The numbers below the ASCL1 blots indicate the values of the bands relative to the scrambled siRNA (siScr) control value after normalization against GAPDH. **d** Quantification of cell viability relative to the siScr and non-doxycycline control cells 5 days after knockdown of endogenous *ASCL1* in Lu139^SCLC-A^ cells with or without NEUROD1 co-expression. **e** Immunoblots showing the expression of ASCL1 and NEUROD1 in H524^SCLC-N^-TetO-ASCL1 cells with (for 5 days) or without doxycycline treatment under activation of endogenous *NEUROD1* by the CRISPRa system. The numbers below the NEUROD1 blots indicate the values of the bands relative to the non-doxycycline CRISPRa vector control value after normalization against GAPDH. **f** Quantification of cell viability 5 days after treatment with doxycycline relative to corresponding non-doxycycline control cells in H524^SCLC-N^ cells co-expressing ASCL1 with NEUROD1 after the CRISPRa-mediated activation of *NEUROD1*. **g** Immunoblots showing the expression of ASCL1, MYC, and NEUROD1 in H524^SCLC-N^-TetO-ASCL1 cells with (for 5 days) or without doxycycline treatment after activation of *MYC* by the CRISPRa system. The numbers below the MYC blots indicate the values of the respective bands relative to the non-doxycycline CRISPRa vector control value after normalization against GAPDH. The numbers below the NEUROD1 blots indicate the values of the bands relative to the corresponding non-doxycycline control values after normalization against GAPDH. **h** Quantification of cell viability 5 days after treatment with doxycycline relative to corresponding non-doxycycline control cells in H524^SCLC-N^ cells co-expressing ASCL1 with NEUROD1 after the CRISPRa-mediated activation of *MYC*. **i** Dose-response analyses for an EZH2 inhibitor, tazemetostat, in Lu139^SCLC-A^-TetO-NEUROD1 cells and H524^SCLC-N^-TetO-ASCL1 cells. Cells were treated with tazemetostat with or without doxycycline for 72 hours. The X-axis indicates drug concentration, and the *Y* axis indicates fluorescence intensity. Cell viability was assessed using alamarBlue Cell Viability Regent (**b**, **d**, **f**, **h**, **i**). Each immunoblot image is a representative of at least two independent procedures (**a**, **c**, **e**, **g**). Error bars indicate ± the standard error of the mean (SEM) from *N* = 3 independent experiments (**b**, **d**, **f**, **h**, **i**). Multi-group comparisons were performed using the one-way ANOVA test (**b**, **f**, **h**) with post-hoc analysis using the Holm method (**d**).

As downregulation of endogenous ASCL1 and NEUROD1 preceded the loss of viability and apoptotic cell death (Fig. 3h, i), we reasoned the repression of ASCL1 in SCLC-A cells and of NEUROD1 in SCLC-N cells to be potential causes of the detrimental effects on co-expression of NEUROD1 and ASCL1, respectively. To address this, we activated *ASCL1* and *NEUROD1* in Lu139^SCLC-A^-TetO-NEUROD1 and H524^SCLC-N^-TetO-ASCL1 cells, respectively, using the CRISPR/dCas9 synergistic activation mediator (SAM) CRISPR activation (CRISPRa) system, which allows efficient induction of endogenous gene expression. However, after activation of endogenous *ASCL1* in Lu139-NEUROD1 cells (Fig. 6a), the reduced cell viability was not significantly rescued (approximately 15%) (Fig. 6b). Reciprocally, knockdown of *ASCL1* using siRNAs (validated in parental Lu139^SCLC-A^ cells) (Fig. 6c) significantly enhanced the decreased cell viability after NEUROD1 co-expression, by ~35% (Fig. 6d). These data indicate that the direct repression of *ASCL1* is only partially involved in the mechanisms by which NEUROD1 co-expression exerts toxic effects in Lu139^SCLC-A^ cells. Similarly, activation of endogenous *NEUROD1* in H524^SCLC-N^-TetO-ASCL1 cells (Fig. 6e) did not significantly rescue the suppression of cell viability caused by ASCL1 co-expression (Fig. 6f). Given the crucial role of MYC in the pathogenesis of SCLC-N cells, we also rescued *MYC* expression in H524^SCLC-N^-TetO-ASCL1 cells (Fig. 6g). Although MYC is known to maintain NEUROD1 expression^15^, *MYC* rescue did not enhance NEUROD1 expression in this *MYC*-amplified cell line in the absence of doxycycline treatment (Fig. 6g), suggesting that *MYC* amplification alone may be sufficient to saturate its regulatory effect on NEUROD1 expression. Furthermore, it did not mitigate the suppression of NEUROD1 induced by ASCL1 co-expression (Fig. 6g). Moreover, activation of *MYC* did not significantly alleviate the detrimental effects of ASCL1 co-expression (Fig. 6h).

Enrichr analysis showed that SUZ12 or EZH2, both components of Polycomb repressive complex 2, were among the top candidate factors that were upregulated or downregulated in Lu139^SCLC-A^-TetO-NEUROD1 and H524^SCLC-N^-TetO-ASCL1 cells (Supplementary Fig. 4f). We therefore next treated these cells with an EZH2 inhibitor, tazemetostat, to examine the involvement of EZH2 in our model. However, EZH2 inhibition did not rescue the reduced viability caused by exogenous expression of the counterpart TFs (Fig. 6i).

### NEUROD1 co-expression in SCLC-A cells causes apoptosis by downregulating anti-apoptotic BCL2 expression

**Fig. 7:**
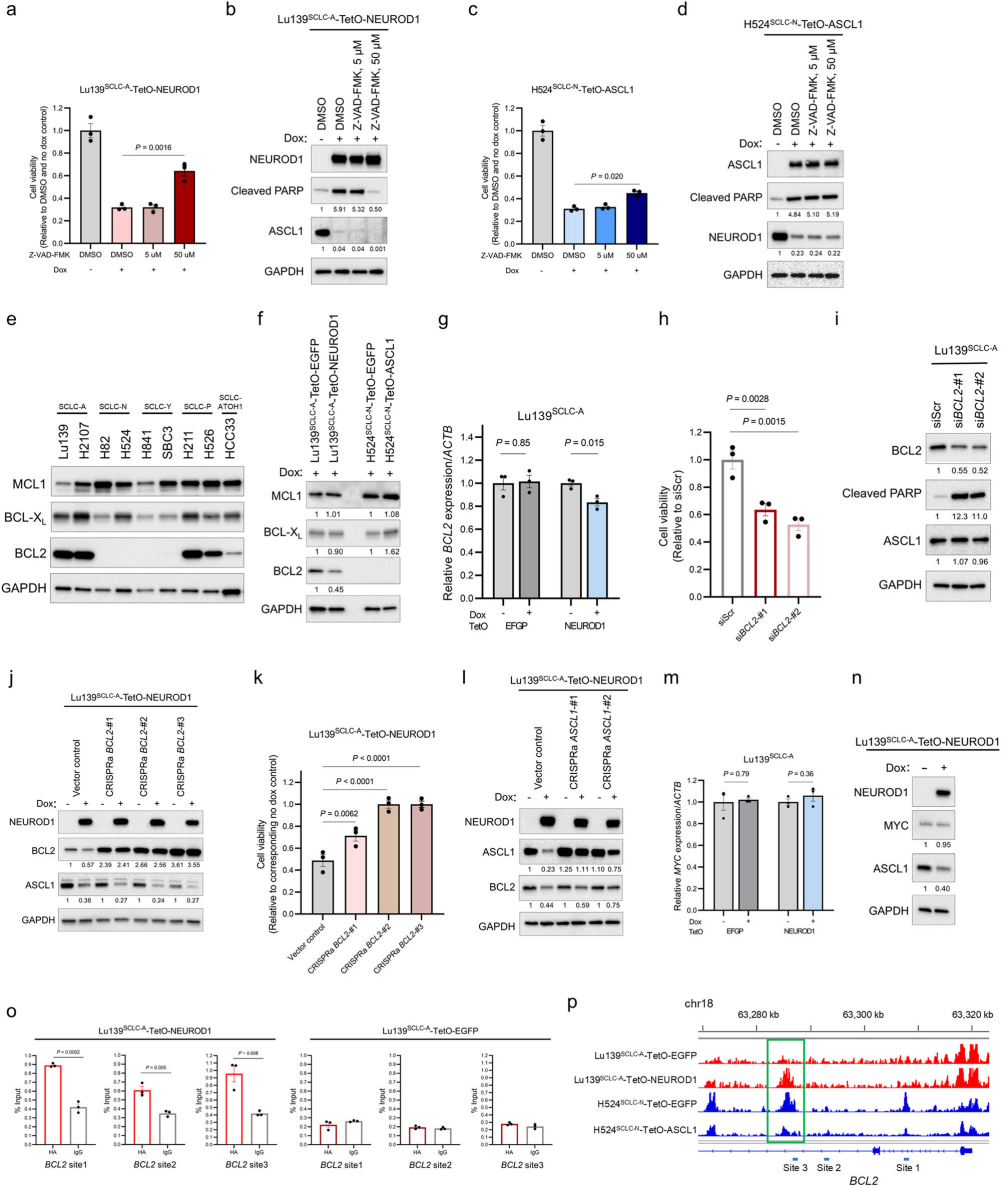
Co-expressed NEUROD1 in SCLC-A cells downregulates BCL2 and causes caspase-dependent apoptotic cell death. **a** Quantification of cell viability relative to non-doxycycline and DMSO-treated control cells 72 hours after treatment with doxycycline and Z-VAD-FMK, a pan-caspase inhibitor, in Lu139^SCLC-A^-TetO-NEUROD1 cells. **b** Immunoblot showing exogenous NEUROD1, endogenous ASCL1, and cleaved PARP after treatment with doxycycline and Z-VAD-FMK for 72 hours in Lu139^SCLC-A^-TetO-NEUROD1 cells. The numbers below the cleaved PARP, and ASCL1 blots indicate the values of the bands relative to the DMSO and no dox control values after normalization against GAPDH. **c** Quantification of cell viability relative to non-doxycycline and DMSO control cells after treatment for 72 hours with doxycycline and Z-VAD-FMK in H524^SCLC-N^-TetO-ASCL11 cells. **d** Immunoblot showing exogenous ASCL1, endogenous NEUROD1, and cleaved PARP after treatment for 72 hours with doxycycline and Z-VAD-FMK in H524^SCLC-N^-TetO-ASCL11 cells. The numbers below the cleaved PARP, and NEUROD1 blots indicate the values of the bands relative to the DMSO and no dox control values after normalization against GAPDH. **e** Immunoblot showing the expression of anti-apoptotic BCL2 family proteins (MCL1, BCL-X_L_, and BCL2) in SCLC cell lines. **f** Immunoblot showing the expression of MCL1, BCL-X_L_, and BCL2 in Lu139^SCLC-A^-TetO-NEUROD1 cells and H524^SCLC-N^-TetO-ASCL1 cells after treatment for 5 days with doxycycline in comparison with the corresponding EGFP-expressing control cells. The numbers below the MCL1, BCL-X_L_, and BCL2 blots indicate the values of the bands relative to the corresponding EGFP-expressing control values after normalization against GAPDH. **g** Expression levels of endogenous *BCL2* transcripts after NEUROD1 co-expression in Lu139^SCLC-A^ cells, relative to non-doxycycline controls after normalization against *ACTB* expression. The EGFP-expressing cells were used as controls. Cells were treated with doxycycline for 72 hours. **h** Quantification of Lu139^SCLC-A^ cell viability 5 days after knockdown of *BCL2* relative to the siScr control cells. **i** Immunoblot showing the expression of BCL2, cleaved PARP, and ASCL1 5 days after knockdown of *BCL2* in Lu139^SCLC-A^ cells. The numbers below the BCL2, cleaved PARP, and ASCL1 blots indicate the values of the bands relative to the scrambled siRNA (siScr) control values after normalization against GAPDH. **j** Immunoblot showing the expression of NEUROD1, BCL2, and ASCL1 in Lu139^SCLC-A^-TetO-NEUROD1 cells with (for 5 days) or without doxycycline treatment after activation of *BCL2* by the CRISPRa system. The numbers below the BCL2 blots indicate the values of the respective bands relative to the non-doxycycline CRISPRa vector control value after normalization against GAPDH. The numbers below the ASCL1 blots indicate the values of the bands relative to the corresponding non-doxycycline control values after normalization against GAPDH. **k** Quantification of cell viability relative to corresponding non-doxycycline control cells in Lu139^SCLC-A^-TetO-NEUROD1 cells with CRISPRa-mediated activation of BCL2 after treatment with doxycycline for 5 days. **l** Immunoblot showing the expression of NEUROD1, ASCL1, and BCL2 in Lu139^SCLC-A^-TetO-NEUROD1 cells with (for 5 days) or without doxycycline treatment after activation of *ASCL1* by the CRISPRa system. The numbers below the ASCL1 blots indicate the values of the respective bands relative to the non-doxycycline CRISPRa vector control value after normalization against GAPDH. The numbers below the BCL2 blots indicate the values of the bands relative to the corresponding non-doxycycline control values after normalization against GAPDH. **m** Expression levels of endogenous *MYC* transcripts after NEUROD1 co-expression for 72 hours in Lu139^SCLC-A^ cells. Values relative to non-doxycycline controls after normalization against *ACTB* expression are shown. The EGFP-expressing cells were used as controls. **n** Immunoblot showing the expression of NEUROD1, MYC, and ASCL1 in Lu139^SCLC-A^-TetO-NEUROD1 cells with or without doxycycline treatment for 5 days. The numbers below the MYC and ASCL1 blots indicate the values of the bands relative to the corresponding non-doxycycline control values after normalization against GAPDH. **o** CUT&RUN assay and quantitative PCR for binding sites of exogenous NEUROD1 on the *BCL2* loci in Lu139^SCLC-A^-TetO-NEUROD1 cells (left). Lu139^SCLC-A^-TetO-EGFP cells were used as a control (right). The anti-HA-tag antibody was used to detect exogenous NEUROD1 and EGFP with C-terminal HA-tags, and rabbit IgG isotype was used as a negative control. The amount of obtained DNA in each sample is represented as a signal relative to the total amount of input chromatin. **p** Representative ATAC-seq (assay for transposase-accessible chromatin with sequencing) data tracks at the *BCL2* gene locus in Lu139^SCLC-A^ cells expressing EGFP or NEUROD1 (red) and H524^SCLC-N^ cells expressing EGFP or ASCL1 (blue). The green square indicates the region in which chromatin accessibility was significantly increased in Lu139^SCLC-A^-TetO-NEUROD1 cells, and that was reciprocally decreased in H524^SCLC-N^-TetO-ASCL1 cells. The CUT&RUN-quantitative PCR primers for site 3 were designed to cover this region. Target sites 1 and 2 analyzed in the CUT&RUN-quantitative PCR assay are also indicated. Error bars indicate ± the standard error of the mean (SEM) from *N* = 3 independent experiments (**a**, **c**, **g**, **h**, **k**, **m**, **o**). Multi-group comparisons were performed using the one-way ANOVA test with post-hoc analysis using the Holm method (**h**, **k**). Each Immunoblot image is representative of at least two independent procedures (**b**, **d**–**f**, **i**, **j**, **l**, **n**). Cell viability was assessed using alamarBlue Cell Viability Regent (**a**, **c**, **h**, **k**).

**Fig. 8:**
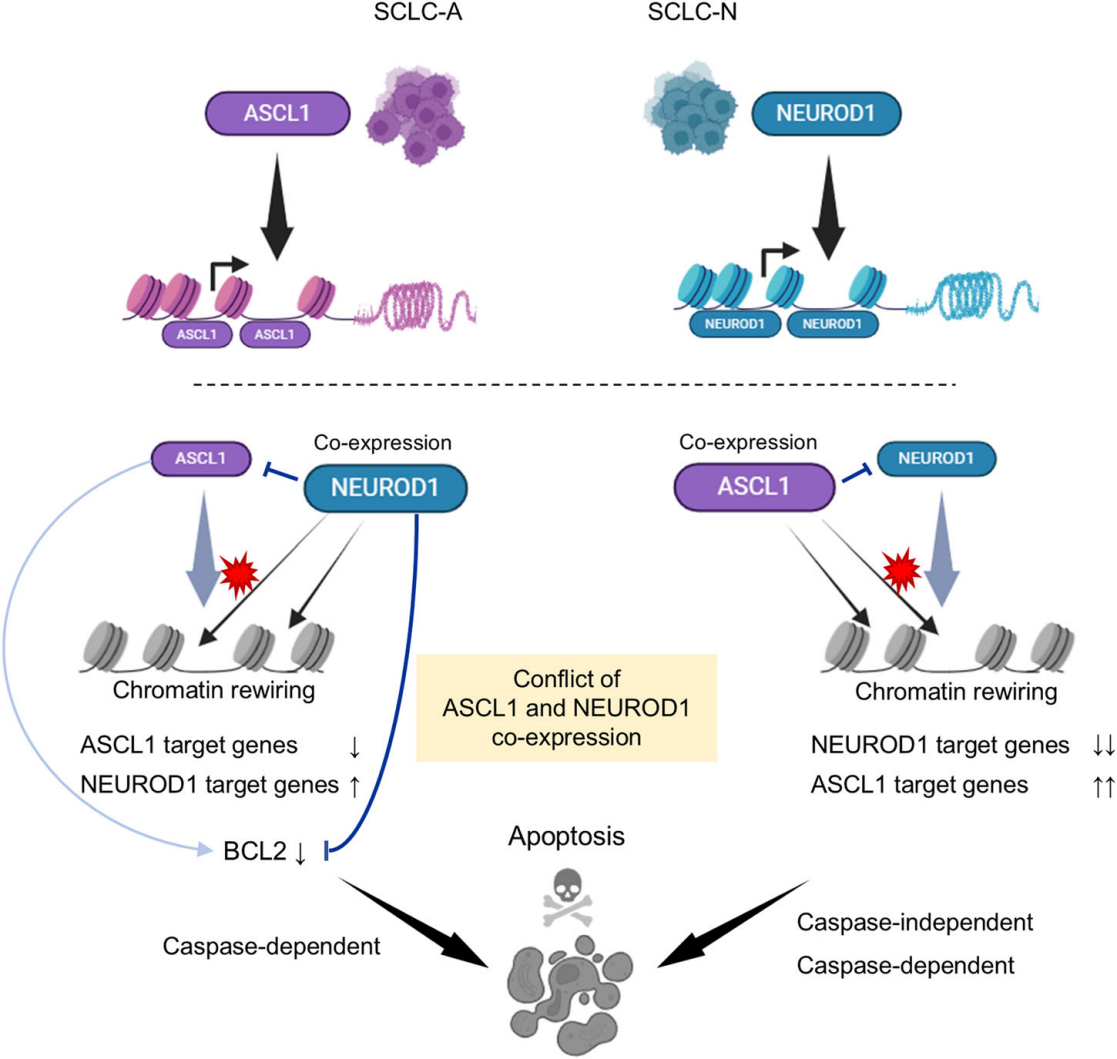
Schematic representation of the mechanisms underlying the toxicity caused by co-expression of ASCL1 and NEUROD1 in SCLC cells. (Top) SCLC-A and SCLC-N cells are primarily driven by the lineage-specific transcription factors, ASCL1 or NEUROD1, respectively. (Bottom) In SCLC-A cells, co-expression of NEUROD1 leads to suppression of ASCL1, downregulation of ASCL1 target genes, and upregulation of NEUROD1 target genes. This process is accompanied by genome-wide changes in chromatin accessibility. NEUROD1 co-expression induces caspase-dependent apoptotic cell death by directly repressing the anti-apoptotic protein, BCL2, with additional repression of its upstream regulator, ASCL1, further reinforcing this effect (left). In SCLC-N cells, co-expressed ASCL1 suppresses NEUROD1 and reciprocally, but with greater efficiency, downregulates NEUROD1 target genes while upregulating ASCL1 target genes, with notable changes in the chromatin accessibility landscape. In these cells, apoptosis induced by ASCL1 co-expression involves both caspase-dependent and -independent mechanisms (right).

We further sought to determine the molecular mechanism behind ASCL1 and NEUROD1 co-expression-mediated apoptosis. A pan-caspase inhibitor, Z-VAD-FMK, significantly rescued decreased cell viability in Lu139^SCLC-A^-TetO-NEUROD1 cells at a concentration of 50 μM (Fig. 7a). While ASCL1 expression remained repressed following this treatment, the cleaved PARP level was completely decreased to that in the non-doxycycline control at 50 μM (Fig. 7b). In contrast, this treatment only modestly rescued reduced viability of H524^SCLC-N^-TetO-ASCL1 cells (Fig. 7c), and it did not reduce the cleaved PARP level (Fig. 7d). These results indicate that caspase-dependent apoptosis is responsible for cell death following co-expression of NEUROD1 in Lu139^SCLC-A^ cells but that it plays little role in the apoptosis caused by ASCL1 co-expression in H524^SCLC-N^ cells. We next focused on anti-apoptotic BCL2 family proteins, including MCL1, BCL-X_L_, and BCL2. Among these, BCL2 was highly expressed in SCLC-A and SCLC-P cell lines but exhibited low expression in HCC33^SCLC-ATOH1^ cells and was absent in SCLC-N and SCLC-Y cell lines (Fig. 7e). Furthermore, BCL2 was downregulated in Lu139^SCLC-A^ cells following NEUROD1 co-expression at the protein (Fig. 7f) and mRNA (Fig. 7g) levels, whereas ASCL1 co-expression did not induce BCL2 expression in H524^SCLC-N^ cells (Fig. 7f). Consistent with previous reports^36,37^ showing that BCL2 inhibition may be relevant to the non-MYC-driven SCLC-A subtype, knockdown of *BCL2* significantly reduced cell viability in Lu139^SCLC-A^ cells (Fig. 7h) and induced apoptosis without affecting ASCL1 expression levels (Fig. 7i). Notably, CRISPRa-mediated activation of *BCL2* in Lu139^SCLC-A^-TetO-NEUROD1 cells (Fig. 7j) significantly, and almost completely, rescued decreased cell viability following NEUROD1 induction (CRISPRa *BCL2*-#2 and -#3; Fig. 7k), without reversing the suppression of ASCL1 by NEUROD1 co-expression. In contrast, the modest rescue of cell viability observed in *ASCL1*-activated CRISPRa Lu139^SCLC-A^-TetO-NEUROD1 cells (Fig. 6b) was consistent with the limited restoration of BCL2 expression (Fig. 7l). Considering a pivotal role of MYC in downregulating *BCL2* through DNA methyltransferase 3a-mediated DNA methylation^36^, we asked whether NEUROD1-mediated downregulation of BCL2 was MYC-dependent. However, MYC was not upregulated at either mRNA (Fig. 7m) or protein (Fig. 7n) level. CUT&RUN-quantitative PCR analyses revealed that NEUROD1 bound to the *BCL2* gene (Fig. 7o). Notably, Lu139^SCLC-A^-TetO-NEUROD1 cells showed significantly increased chromatin accessibility at *BCL2* site 3 in the CUT&RUN-quantitative PCR analysis, while H524^SCLC-N^-TetO-ASCL1 cells showed significantly decreased chromatin accessibility (Fig. 7p). Collectively, these findings indicate that co-expressed NEUROD1 in Lu139^SCLC-A^ cells binds to the *BCL2* gene and suppresses its expression without altering MYC expression levels. Furthermore, the direct repression of BCL2 by NEUROD1 is further enhanced through NEUROD1-mediated downregulation of ASCL1, a crucial regulator of BCL2^29,37^. This regulatory process ultimately leads to apoptotic cell death (Fig. 8).

## Discussion

Inspired by the mutually exclusive expression patterns of lineage-specific TFs reported in SCLC, we explored here whether any combination of TFs could exert selective lethality in SCLC cells. We showed that the two most prevalent TFs in SCLC, ASCL1 and NEUROD1, which together are expressed in 70%–85% of SCLC cases^8,12^, demonstrated strong mutual exclusivity at a cellular resolution. Consistent with this notion, co-expression of ASCL1 and NEUROD1 in both SCLC-A and SCLC-N cell lines caused growth inhibition and apoptosis. Mechanistically, co-expression of ASCL1 or NEUROD1 in a SCLC-N or SCLC-A cell line, respectively, caused transcriptional reprogramming with suppression of the cell fate defined by the endogenous TF and the alternative upregulation of gene expression programs driven by the ectopically expressed TF, accompanied by marked changes in the genome-wide chromatin accessibility. Furthermore, co-expressed NEUROD1 in Lu139^SCLC-A^ cells downregulated a crucial anti-apoptotic protein, BCL2, which was responsible for apoptotic cell death in our model.

Previous studies have reported the existence of intra-tumoral heterogeneities in the expression of these lineage-specific TFs, particularly co-expression, in clinical SCLC tumor samples and mouse models^8,9,38–40^, which contradicts our results. However, the reported co-expression of these TFs was based on IHC^8,9,39,40^ and bulk RNA-seq analyses^38^, which will not accurately represent the TF expression status at the level of single cells. Moreover, given the temporal evolution from SCLC-A to SCLC-N, observed in both the MYC-driven SCLC model^11^ and the KDM6A-inactivation mouse model^13^, it is possible that these observed intra-tumoral heterogeneities are snapshots of cells at different stages of subtype switching. Indeed, spatially distinct regions of SCLC-A and SCLC-N cells within a single tumor have been observed in both the present and previous studies^12^. In addition, our double-staining immunofluorescence showed a high degree of mutual exclusivity of cellular ASCL1 and NEUROD1 expression, in keeping with previous reports of SCLC^7,13,14,41^ and neuroendocrine carcinomas arising at different anatomic sites^42^.

Our results demonstrated that ASCL1 co-expression in SCLC-N cell lines caused more striking changes in chromatin accessibility and gene expression profiles than NEUROD1 co-expression in SCLC-A cell lines. This could account for the consistent growth suppression observed in the SCLC-N cell lines (H524 and H82) following ASCL1 co-expression, while the viability of the H2107 SCLC-A cell line was not significantly affected by NEUROD1 co-expression, despite the significant downregulation of endogenous ASCL1. These results suggest that there is a higher barrier to a lineage shift from SCLC-N to SCLC-A than to a shift from SCLC-A to SCLC-N, which is supported by previous studies showing unidirectional plasticity from SCLC-A to SCLC-N^11–15^. Therefore, although the precise mechanism underlying co-expressed ASCL1-mediated cell death in SCLC-N cells remains to be elucidated (Fig. 8), emerging technologies such as nanomedicine^43^ and RNA therapeutics^44^ offer the potential to induce ASCL1 co-expression selectively in SCLC-N cells, presenting a more promising therapeutic strategy compared to the induction of NEUROD1 co-expression selectively in SCLC-A cells. This counterintuitive treatment approach could not only lead to selective tumor cell killing in one subtype but also enhance the efficacy of future lineage-specific precision therapies for cancer cells in the other subtype by targeting tumor heterogeneity.

ASCL1 and NEUROD1, which define the SCLC lineages showing neuroendocrine differentiation, are both class II bHLH TFs characterized by their tissue-specific expression profiles^45^. Both TFs are involved in the process of central nervous system development and differentiation^45^. During embryogenesis, multiple bHLH TFs are expressed in distinct regions in the telencephalon, spinal cord, and retina^45^. Moreover, during development, neocortical neurons are generated sequentially from multipotent progenitors through a series of identity transitions. This neuronal phenotype specification requires a genetic cascade in which the bHLH TFs NEUROGENIN1 and NEUROGENIN2 repress ASCL1 to inhibit differentiation into the subcortical neuronal phenotype^46,47^. These spatially and temporally distinct expression features of bHLH TFs in central nervous system development resemble those observed in SCLC, suggesting the disadvantages for both neuronal and SCLC cells of retaining the dual lineages in one cell.

Transcriptional addiction is one of the hallmarks of SCLC^48^. Surprisingly, up to 60% of the RNA transcripts in SCLC cell lines are synthesized from ASCL1 and/or NEUROD1 target genes^49^, highlighting the crucial roles of these TFs as master regulators. Although both ASCL1 and NEUROD1 bind DNA sequences containing similar E-box motifs, these TFs preferentially bind distinct E-box motifs, and regulate mostly distinct genes in the SCLC cell lines driven by each TF, due, at least in part, to the cooperation of cell line- or cell lineage-specific co-factors^29^. Of note, there are also significant differences in the chromatin landscape between SCLC subtypes, and the molecular SCLC classification can be predicted by super-enhancer signatures^50^. Because both ASCL1 and NEUROD1 are pioneer TFs^31–34^, we reason that the lethal effects of ASCL1 and NEUROD1 co-expression in our model are likely to result from the cellular unfitness for acute lineage conversion, coupled with the pronounced changes in chromatin accessibility driven by the ectopically expressed TFs. Nonetheless, it remains to be established how ASCL1 and NEUROD1 can remodel chromatin to shut down the expression program of their counterpart gene. Interestingly, in the mouse adult epidermal stem cell model, another pioneer factor, SOX9, silences the epidermal fate during fate conversion by competing away co-factors and other TFs from active epidermal enhancers^51^. In hepatobiliary reprogramming, the hepatocyte phenotype is repressed by the pioneer factor, SOX4, which binds hepatocyte enhancers through recognition of the shared motif sequences and evicts previously bound hepatocyte TFs, such as HNF4A^52^. In the present study, we show that ASCL1 and NEUROD1 can bind to each other’s gene loci and that NEUROD1 can also directly bind to the *BCL2* gene, which is known to act downstream of ASCL1^29,37^. Interestingly, chromatin accessibility at one of the NEUROD1-binding *BCL2* loci (site 3 in the CUT&RUN analysis) showed reciprocal changes between Lu139^SCLC-A^-TetO-NEUROD1 cells and H524^SCLC-N^-TetO-ASCL1 cells after doxycycline treatment (Fig. 7p), suggesting competitive regulation of *BCL2* between ASCL1 and NEUROD1. Ectopically expressed NEUROD1-mediated apoptosis through downregulation of *BCL2* was also reported in microglia^53^. Further studies are required to elucidate the regulatory mechanisms underlying the mutual repression of ASCL1 and NEUROD1 along with the direct NEUROD1-mediated repression of BCL2 across different types of cancer and disease.

Previous studies have speculated that YAP1 is a transcriptional driver for a rare non-neuroendocrine subtype of SCLC^5,6,9^ that may emerge during disease evolution^11^. It has also been suggested that an unbiased hierarchical clustering approach may fail to detect the SCLC-Y subtype^7^, as YAP1 is expressed in a subpopulation of SCLC cell clusters^54,55^, representing an intra-tumoral heterogeneity. Nonetheless, we identified SCLC-Y in a minority (6.6%) of clinical SCLC specimens and found that overexpression of activated YAP1 in other SCLC cell line subtypes exerted certain detrimental effects. However, we did not focus on YAP1 in this study, because multiple recent studies have questioned the value of YAP1 as a robust subtype-defining factor in SCLC^8,10,56^.

Our data suggest that ATOH1 may cause lineage conflict and synthetic lethality when co-expressed with NEUROD1. However, the rarity of ATOH1-driven SCLC and the unavailability of high-quality anti-ATOH1 antibodies have precluded intense research. Future studies may provide additional insights into this relatively unstudied TF in SCLC.

A limitation of this study is that we focused on the cancer-cell-intrinsic effects of lineage-specific TF co-expression, but not on the impact of TF co-expression on immune evasion in our exploration of the mechanisms underlying their mutual exclusivity. However, although immunotherapy plays an important role in the treatment of SCLC, considering that both SCLC-A and SCLC-N subtypes have been associated with limited benefit from immune checkpoint blockade therapy compared with the SCLC-inflamed subtype^12^, exploration of the consequences of ASCL1 and NEUROD1 co-expression in SCLC cells in the context of antitumor immunity was less of a priority.

In conclusion, among the SCLC lineage-defining TFs, we have demonstrated that ASCL1 and NEUROD1 mutually silence cell fate by reprograming the chromatin accessibility and that co-expressed NEUROD1 in ASCL1-driven SCLC cells can cause apoptosis through downregulation of BCL2. Dual expression of these TFs in an SCLC cell is therefore toxic, likely by disrupting the cell differentiation integrity and competitive regulation of important target genes. This provides the biological basis for the observation that subtype switching is preferential in SCLC, as opposed to co-expression of both TFs. Further studies are warranted to explore this previously unappreciated vulnerability of SCLC to establish a novel therapeutic strategy.

## Methods

### Study overview

This study was approved by the institutional review boards at Hamamatsu University School of Medicine (#20-259) and four hospitals in Japan. Patient approval and written informed consent were waived because this study was based on reviews of previously acquired patient samples and records. This information was provided on the website (https://www.hama-med.ac.jp/research/clinical-res/erc/disclosure-info/index.html). All analyses were conducted in compliance with the ethical standards of the Declaration of Helsinki. Research involving recombinant DNA was conducted under Biosafety Level 2 containment and was approved by the Hamamatsu University Recombinant DNA Experiment Safety Committee (#2-27), in accordance with the National Institutes of Health Guidelines for Research Involving Recombinant DNA Molecules.

### Patient samples

Formalin-fixed and paraffin-embedded tumor tissue blocks of resected SCLC were collected from Hamamatsu University Hospital and four cooperating hospitals in Japan. Surgery was performed between January 2000 and December 2020. Tissue cores were punched out using cylinders with a diameter of 3 mm (Azumaya, Tokyo, Japan) and were aligned to make tissue microarrays (TMAs) as described previously^57^.

### Immunohistochemical analysis

TMA sections were used for IHC as described previously^58^. All the TMA cores were confirmed to contain a sufficient number of tumor cells on hematoxylin–eosin staining sections. Primary antibodies were used against ASCL1 (1:100 dilution, 24B72D11, BD Biosciences, San Jose, CA), NEUROD1 (1:1000 dilution, EPR20766, Abcam, Cambridge, UK), POU2F3 (1:200 dilution, polyclonal, Novus Biologicals, Centennial, CO), YAP1 (1:400 dilution, D8H1X, Cell Signaling Technology [CST], Danvers, MA), Rb (1:1000 dilution, 4H1, CST), and p53 (1:200 dilution, 7F5, CST). The nuclear expression levels of each protein were assessed based on the H-score, which was calculated by multiplying the staining intensity (scored on a scale of 0 [absent], 1 [weak], 2 [moderate], or 3 [strong]) by the percentage of the stained tumor area (0%–100%), and the total H-scores ranged from 0 to 300. Protein expression was considered positive if the H-score was >10^8,9^. The dominant TF was determined as that with the highest H-score of the four factors^9^. An H-score of 0 for Rb indicated the inactivation of Rb^59^. Intense p53 staining in >80% of the tumor cells (overexpression pattern) and complete loss of p53 staining (absent pattern) indicated p53 inactivation, whereas heterogeneous nuclear p53 staining was considered WT^59,60^.

### Immunofluorescence analysis

TMA sections were subjected to deparaffinization, rehydration, and antigen retrieval. After blocking, the sections were incubated with anti-ASCL1 (1:50 dilution, 24B72D11, BD Bioscience) and anti-NEUROD1 (1:200 dilution, EPR20766, Abcam) antibodies. SCLC cells cultured in suspension as floating clusters were concentrated on glass slides using a Cytospin 4 cytocentrifuge (Thermo Fisher Scientific). After fixation with 4% paraformaldehyde, blocking and incubation with primary antibodies were similarly performed. Alexa Fluor-488 goat anti-mouse IgG and Alexa Fluor-546 goat anti-rabbit IgG (Thermo Fisher Scientific, Waltham, MA) were used as the secondary antibodies. Nuclei were stained with DAPI. Multispectral images were obtained by scanning the stained slides with a TSC SP8 confocal microscope (Leica, Wetzlar, Germany).

### Cell lines and culture conditions

The human SCLC cell lines H2107, H82, H526, and H524 were a generous gift from Dr. William Lockwood at BC Cancer Agency (Vancouver, BC, Canada). NCI-H841 (H841) and H211 cells were obtained from the American Type Culture Collection (ATCC; Manassas, VA). Lu135, Lu139, and Lu165 cells were obtained from RIKEN BRC (Tsukuba, Japan) through the National BioResource Project of the MEXT, Japan. SBC3 cells and HCC33 cells were obtained from the Japanese Collection of Research Bioresources (Osaka, Japan) and the Leibniz Institute DSMZ-German Collection of Microorganisms and Cell Cultures GmbH (Braunschweig, Germany), respectively. Among the 11 SCLC cell lines used in this study, H2107, Lu139, H82, H524, H526, HCC33, and Lu165 cells grow in suspension; Lu135 cells grow in a mixture of adherent and suspended cells; H211 cells are loosely adherent; and H841 and SBC3 cells are adherent. All the SCLC cell lines, except for SBC3, harbor *TP53* mutations and eight (H2107, Lu139, H82, H524, Lu135, H526, HCC33, and Lu165) harbor inactivated Rb by either mutations or loss. Lenti-X 293 T cells were obtained from TAKARA (Kusatsu, Japan). All cell lines except for H2107 and Lenti-X 293 T cells were cultured in RPMI 1640 medium (Thermo Fisher Scientific) supplemented with 10% fetal bovine serum (FBS; Sigma-Aldrich, St. Louis, MO). H2107 cells were cultured in DMEM/F-12 medium (Thermo Fisher Scientific) supplemented with 5% FBS. Lenti-X 293 T cells were cultured in DMEM (Thermo Fisher Scientific) with 10% FBS. Cells were maintained in a 5% CO_2_ and 95% air incubator at 37 °C.

### Chemicals

A pan-caspase inhibitor, Z-VAD-FMK, and a proteasome inhibitor, MG132, were purchased from Selleck Chemicals (Houston, TX). An EZH2 inhibitor, tazemetostat, was purchased from MedChemExpress (Monmouth Junction, NJ).

### Plasmids and generation of Tet-On stable cell lines overexpressing ASCL1, NEUROD1, POU2F3, ATOH1, YAP1, and EGFP

pENTR221_ASCL1 (#100009031), pENTR221_NEUROD1 (#100009207), pENTR221_POU2F3 (#1000070023), and pENTR221_ATOH1 (#1000070300) were obtained from DNAFORM (Yokohama, Japan). pDONR221_EGFP (#25899) and pENTR-3xFLAG-YAP1^S127A^ (#42239) were obtained from Addgene (Cambridge, MA). Full-length *ASCL1*, *NEUROD1*, *POU2F3*, and *YAP1*^S127A^ were cloned into the Tet-inducible lentiviral vector pInducer20 (#44012; Addgene) that provides the C-terminal HA-tag using Gateway LR Clonase II enzyme mix (Thermo Fisher Scientific). Lentiviral supernatants were produced and SCLC cell lines (Lu139, H2107, H524, H82, H526, SBC3, and HCC33) were infected with lentivirus as described previously^28,61^. After transduction, stable polyclonal cells were selected with geneticin (Thermo Fisher Scientific) at 1500 µg/mL for two weeks. Doxycycline (FUJIFILM, Tokyo, Japan) was added at 100 ng/mL to induce gene expression unless otherwise mentioned.

### Immunoblotting

Cell lysates were prepared and subjected to immunoblot analysis as previously described^61^ using antibodies against GAPDH (Santa Cruz Biotechnology, Dallas, TX), ASCL1 (24B72D11, BD Biosciences), NEUROD1 (D35G2, CST), YAP1/TAZ (D24E4, CST), YAP1 (D8H1X, CST), POU2F3 (polyclonal, Sigma-Aldrich), ATOH1 (polyclonal, Proteintech, Rosemont, IL), MYC (polyclonal, Santa Cruz Biotechnology), cleaved PARP (Asp214, CST), BCL2 (D17C4, CST), MCL1 (D35A5, CST), BCL-X_L_ (54H6, CST), E-cadherin (24E10, CST), N-cadherin (D4R1H, CST), ZO-1 (D7D12, CST), Vimentin (D21H13, CST), Snail (C15D3, CST), Slug (C19G7, CST), phospho-histone H2A.X (Ser139; 20E3, CST), Cyclin B1 (polyclonal, CST), and Cyclin D1 (92G2, CST).

### RNA isolation and quantitative PCR

Total RNA was extracted using an RNeasy Plus Mini Kit (#74136, QIAGEN, Hilden, Germany) according to the manufacturer’s instructions. Complementary DNA was synthesized from the total RNA using a High-Capacity RNA-to-cDNA Kit (Thermo Fisher Scientific). Quantitative PCR reactions were performed on a StepOnePlus Real-Time PCR System (Thermo Fisher Scientific) using TaqMan Gene Expression Assay Mix (Thermo Fisher Scientific) for *ASCL1* (Hs00269932_m1), *NEUROD1* (Hs00159598_m1), *MYC* (Hs00153408_m1), and *BCL2* (Hs00608023_m1). The relative RNA expression levels represent an average of triplicates calculated using the ΔΔCt method, with the levels normalized to *ACTB* (Hs999999903_m1).

### Gene silencing

Silencer Select Pre-designed siRNA for *ASCL1* (s1656 and s1658, Thermo Fisher Scientific), *NEUROD1* (s9458 and s9459, Thermo Fisher Scientific), *BCL2* (s1915 and s1916, Thermo Fisher Scientific), and Silencer Select Negative control (4390843, Thermo Fisher Scientific) were used for RNA interference. Cells (1.0 × 10^6^ cells) were transfected with 5 nM (for si*ASCL1* or si*BCL2*) or 25 nM (for si*NEUROD1*) siRNA using Opti-MEM I Reduced Serum Medium (Thermo Fisher Scientific) and Lipofectamine RNAiMAX Reagent (Thermo Fisher Scientific). The cells were used for further assays 5 days after transfection.

### Cell proliferation assay

To determine the cell viability on day 5 for Lu139, H2107, H524, H82, H526, HCC33, and SBC3 derivatives, cells with doxycycline-inducible constructs were seeded in triplicate in six-well plates with or without doxycycline, at 1.0 × 10^5^ (H2107 and HCC33 derivatives), 5.0 × 10^4^ (Lu139, H524, H82, and H526 derivatives), and 2.5 × 10^4^ (SBC3 derivatives) cells/well. For Lu139-TetO-NEUROD1 cells and H524-TetO-ASCL1 cells, changes in cell viability over time for up to 5 days were also determined. alamarBlue Cell Viability Reagent (Thermo Fisher Scientific) was added, and the fluorescence intensities were measured using Synergy H1 with Gen5 software version 2.09 (BioTek Instruments, Inc, Winooski, VT). Alongside the cell viability, the viable cell numbers were counted using Trypan Blue (Thermo Fisher Scientific) and the automated cell counter Countess II FL (Thermo Fisher Scientific).

### Dose-response analysis

Lu139 and H524 derivative cells were seeded in 96-well plates at a density of 2 × 10^3^ cells/well with or without doxycycline. After incubation for 72 hours with tazemetostat at different concentrations ranging from 0.1 nM to 10 µM, cell viability was assessed using alamarBlue Cell Viability Regent (Thermo Fisher Scientific).

### Cell cycle assay

Cell Cycle Assay Solution Blue (DOJINDO, Kumamoto, Japan) was used for cell cycle assays according to the manufacturer’s instructions. Briefly, Lu139 derivatives and H524 derivatives with or without 5 days of doxycycline treatment were collected, washed with PBS, and incubated with cell cycle assay solution. Thereafter, the DNA content was determined using a Gallios flow cytometer (Beckman Coulter, Miami, FL), followed by analysis using FlowJo software, version 7.6 (Becton, Dickinson and Company, Franklin Lakes, NJ).

### Measurement of apoptosis

An Annexin V-FITC Apoptosis Detection Kit (NACALAI TESQUE, Kyoto, Japan) was used to detect apoptosis by measuring the annexin V- and propidium iodide (PI)-positive cells, according to the manufacturer’s instructions. Lu139 derivatives and H524 derivatives with or without 5 days of doxycycline treatment were collected. EGFP cells were not suitable for this assay because of the overlapping emission wavelengths of FITC and EGFP. The cells were collected, washed with PBS, and resuspended in Annexin V Binding Solution (1 × 10^6^ cells/ml). Then, 5 µL of annexin V-FITC solution and 5 µL of PI solution were added to 100 µL of cell suspension, and the number of apoptotic cells was calculated using a Gallios flow cytometer (Beckman Coulter).

### Microarray gene expression analysis

RNA was extracted from Lu139-TetO-NEUROD1, Lu139-TetO-EGFP, H524-TetO-ASCL1, and H524-TetO-EGFP cells with or without 72 hours of doxycycline treatment (each in triplicate). The RNA samples were used for global gene expression profiling on a Clariom S Assay (human) (Thermo Fisher Scientific) according to the manufacturer’s protocols using a GeneChip Scanner 3000 7 G (Thermo Fisher Scientific). Raw expression data were normalized by the Signal Space Transformation-Robust Multiarray Analysis method. After normalization, each gene expression value was divided by the median expression value of each gene, and these were log_2_ transformed for the following comparative analysis. Significant differentially expressed genes were defined by the criterion of a false discovery rate [FDR]-adjusted *P* value < 0.05 by the Benjamini and Hochberg method. We first performed two-group comparisons of the Lu139-TetO-NEUROD1 and H524-TetO-ASCL1 cells between conditions with and without doxycycline treatment using Student’s t-test. We then determined genes with expression levels that changed significantly between the presence and absence of doxycycline in the corresponding EGFP control cells, as this represents non-specific or doxycycline-mediated changes to the transcriptome. These genes were removed from both the Lu139-TetO-NEUROD1 and H524-TetO-ASCL1 datasets. GSEA was performed using GSEA software version 4.2.3. Default parameters regarding the hallmark gene sets (h.all.v2023.2.Hs.symbols.gmt) were obtained from the Human Molecular Signatures Database (https://www.gsea-msigdb.org/gsea/msigdb/collections.jsp), and previously published gene set lists of ASCL1 or NEUROD1 target genes^29^ were used. Significantly (FDR-adjusted *P* value < 0.05) upregulated or downregulated genes in Lu139-TetO-NEUROD1 cells and H524-TetO-ASCL1 cells compared with the corresponding non-doxycycline control cells were analyzed by Enrichr^62^ (https://maayanlab.cloud/Enrichr/) to identify enriched ENCODE and ChEA consensus tissue factors from the ChIP-X database.

### Proximity ligation assay

A proximity ligation assay was carried out using Duolink flowPLA Detection Kit Green (Sigma-Aldrich) following the manufacturer’s instructions. Briefly, 1.0 × 10^5^ of Lu139-TetO-NEUROD1 cells with or without doxycycline were fixed with 4% paraformaldehyde and permeabilized with 0.1% Triton X-100 in PBS. Cells were blocked with Duolink Blocking Solution (Sigma-Aldrich). After that, cells were incubated with anti-ASCL1 (24B72D11, BD Biosciences) and anti-NEUROD1 (EPR20766, Abcam) antibodies at 1:50 dilution, followed by an incubation with secondary Duolink plus and minus antibodies (Sigma-Aldrich). The PLA probes were ligated in a ligation buffer and amplified in a polymerase mixture. The resuspended cells were sorted using a Gallios flow cytometer (Beckman Coulter, Miami, FL) and analyzed with FlowJo software, version 7.6. The combination of ASCL1 and PROX1 (anti-PROX1 antibody, polyclonal, Proteintech) was set as a positive control^50^.

### CRISPR activation

Single-guide RNA (sgRNA) sequences targeting the promoters of *ASCL1* (sg*ASCL1*-#1, 5′-CTCCCCGCTGCTGCAGCGAGA-3′; sg*ASCL1*-#2, 5′-GCAGCCGCTCGCTGCAGCAG-3′), *NEUROD1* (sg*NEUROD1*-#1, 5′-CCGCTAGCTGAGGGGCTAGC-3′; sg*NEUROD1-*#2, 5′-AGAACGGGGAGCGCACAGCC-3′), *MYC* (sg*MYC*-#1, 5′-GAGAAGCCCTGCCCTTCTCG-3′; sg*MYC*-#2, 5′-CTCCCCTCCTGCCTCGAGAA-3′), and *BCL2* (sg*BCL2*-#1, 5′-TGCTACGAAGTTCTCCCCCC-3′; sg*BCL2*-#2, 5′-CCTCTTCCGCTGCACCCCAC-3′; sg*BCL2*-#3, 5′-AGAAAGGGTGCGCAGCCCGG-3′) were designed using the sgRNA design tool (https://portals.broadinstitute.org/gpp/public/) and cloned into the lentiSAMv2 plasmid (#75112; Addgene). Target cells were transduced with lentiviral particles of lentiMPHv2 (#89308; Addgene) followed by hygromycin selection (400 µg/ml). Stable cells were then transduced with lentiviral particles of the recombinant lentiSAMv2 followed by blasticidin selection (10 µg/ml). Empty lentiSAMv2 was used as a control.

### ATAC-seq analysis

Lu139-TetO-NEUROD1, Lu139-TetO-EGFP, H524-TetO-ASCL1, and H524-TetO-EGFP cells were treated with doxycycline for 72 hours and collected at 2.0 × 10^5^ cells/tube in duplicate at 4°C. ATAC-seq was performed using an ATAC-Seq Kit (Active Motif, Carlsbad, CA) according to the manufacturer’s protocol. In brief, cells were washed and lysed in ATAC Lysis Buffer and centrifuged at 4 °C. After supernatant removal, tagmentation of nuclei was performed using Tagmentation Master Mix containing Tn5 transposons. After incubation, DNA Purification Binding Buffer and 3 M sodium acetate were added and purified using DNA purification columns. PCR amplification of the tagmented DNA was performed using a combination of i7 and i5 indexing primers. The PCR products were cleaned up using SPRI beads. The samples were subsequently sequenced on a NovaSeq 6000 (Illumina, San Diego, CA). The quality of the raw paired-end sequence reads was checked using FastQC^63^ version 0.11.7, and these files were trimmed to remove Illumina Nextera adapter sequences by Skewer^64^ version 0.2.2. The trimmed reads were aligned to the reference genome (human genome assembly hg38) using Bowtie2^65^ version 2.3.4.2. Then, sequence reads mapped to the mitochondrial chromosome and blacklist regions were removed by Samtools^66,67^ version 1.9, and PCR duplicates were removed by Picard version 2.18.11 (http://broadinstitute.github.io/picard/). Peak calling was carried out using MACS2^68^ version 2.1.2 with the parameters “—shift-75 –extsize 150 – nomodel -q 0.01”, and the called peaks from all samples were merged with Bedtools^69^ version 2.27.1. A matrix of peak counts was created with featureCounts^70^ version 1.6.3. The raw read counts were normalized by the Trimmed Mean of the M-values. Principal component analysis (PCA)^71^ of the normalized counts was conducted, and each sample was shown on the 2D dimension of the first and second PCA axes using stats version 3.6.1 and gplots version 3.0.1.1 R packages. Differential analyses were performed using edgeR^72,73^ version 3.26.8. Differential peak regions were detected with the threshold of log_2_(fold change) >1 and FDR-adjusted *P* value < 0.05 by the Benjamini and Hochberg method, and differential peak regions were shown as a heatmap. Signal aggregation plots and a signal heatmap were drawn using deeptools^74^ version 3.2.1. Enriched known motif finding of the differential peak regions was performed by the findMotifs tool in HOMER^75^ version 4.1 with the threshold of log_2_(fold change) >1. Motif enrichment lists in regions of lost and increased accessibility were ranked by *P* values. Genome tracks were visualized by Integrative Genomics Viewer^76^ version 2.16.2 with the hg38 human reference genome. A volcano plot of the differential expression analysis of the microarray data was integrated with the ATAC-seq data. Genes near the differential peaks detected by ATAC-seq when comparing Lu139-TetO-NEUROD1 with Lu139-TetO-EGFP cells or H524-TetO-ASCL1 with H524-TetO-EGFP cells were nominated with the threshold of log_2_(fold change) >1. Among these genes, ASCL1- or NEUROD1-related genes^29^ showing significant differences in gene expression were selected and highlighted on the volcano plots, defined by log_2_(fold change) >1 and FDR *P* value < 0.05.

### CUT&RUN

The CUT&RUN Assay Kit (CST) was used according to the manufacturer’s instructions. Briefly, after doxycycline treatment for 72 hours, Lu139 and H524 derivatives (5.0 × 10^5^ cells for each reaction) were harvested, washed, and bound to activated concanavalin A-coated magnetic beads and permeabilized with wash buffer containing 0.05% digitonin. The bead–cell complex was incubated overnight with the respective antibody at 4 °C. Rabbit IgG isotype control (DA1E, CST) was used for negative control at a dilution of 1:20. To detect exogenous ASCL1, NEUROD1, and EGFP, an anti-HA-Tag antibody (C29F4, CST) was used at a dilution of 1:50. The bead–cell complex was washed and resuspended in pAG/MNase and incubated for 1 hour. After washing, 2 mM CaCl_2_ was added to the cell-bead slurry to initiate digestion, and incubated for 30 minutes at 4°C. The reaction was stopped with the addition of a Stop Buffer containing 20 mM EDTA, 0.05% digitonin, 5 mg/mL RNase A, and 2 pg/mL spike-in DNA. Spike-in DNA was used for sample normalization. Finally, fragments were released by incubation for 30 min at 37 °C. After centrifugation, the supernatant was recovered, and DNA was purified using DNA Purification Buffers and Spin Columns. Finally, binding of ASCL1, NEUROD1, and EGFP onto the *NEUROD1* locus (for ASCL1 and EGFP), and onto the *ASCL1* or *BCL2* loci (for NEUROD1 and EGFP) were quantified by quantitative PCR using THUNDERBIRD SYBR qPCR Mix (TOYOBO, Osaka, Japan). The following primers were used to detect the *ASCL1* locus (*ASCL1* site 1, F: 5′-CCGCAGCCTGTTTCTTTG-3′ and R: 5′-GCTGTCGCTTGACTTGCTT-3′; *ASCL1* site 2, F: 5′-CGGCCAACAAGAAGATGAGT-3′ and R: 5′-TGGAGTAGTTGGGGGAGATG-3′), the *BCL2* locus (*BCL2* site 1, F: 5′-CATCCGTTAGCATGAAGCAA-3′ and R: 5′-AGCCCCTGGAGAAGTATGGT-3′ ; *BCL2* site 2, F: 5′-CTGCCAGCTCCAAGCATAGT-3′ and R: 5′-CCTCTCATGCAACAGTTCAGC-3′; *BCL2* site 3, F: 5′-CAACTGCCACAAGAAGCAAA-3′ and R: 5′-GGCACTCATTCAACAGCAAA-3′), and the *NEUROD1* locus (*NEUROD1* site 1, F: 5′-GTCCGCGGAGTCTCTAACTG-3′ and R: 5′-CATGCGCCATATGGTCTTC-3′; *NEUROD1* site 2, F: 5′-CCCGACACCCCACTCCTA-3′ and R: 5′-TCACAGGGCCAAGATAAAGC-3′).

### Statistical analysis

Differences in the cell experiments were analyzed using Student’s t-test or one-way ANOVA test followed by Holm’s multiple comparisons post-test. All values were analyzed using EZR^77^ version 1.61 (Saitama Medical Center, Jichi Medical University, Saitama, Japan) and GraphPad Prism version 9.5.1 (GraphPad Software, San Diego, CA). *P* values < 0.05 were considered statistically significant. Data are presented as mean ± standard error of the mean of three independent experiments.

## Supplementary information

Supplementary Information

## Data Availability

The datasets generated and/or analyzed during the current study are available in the NCBI GEO repository (GSE269024 and GSE269424).
