# Supplementary material for: Lethal co-expression intolerance underlies the mutually exclusive expression of ASCL1 and NEUROD1 in SCLC cells
Source: NPJ Precis Oncol. 2025 Mar 13;9:74. doi: 10.1038/s41698-025-00860-6 (PMC11906894; doi:10.1038/s41698-025-00860-6)
Supplement: Supplementary file 1 — Supplementary Information [file 41698_2025_860_MOESM1_ESM.docx]

**Supplemental Information File**

**Supplementary Table 1. Patient and tumor characteristics**

|  | *N* = 151 |
| --- | --- |
| **Age (years)** | 72 (52－94) |
| **Sex** |  |
| Male | 138 (91.4) |
| Female | 13 (8.6) |
| **Smoking status** |  |
| Never | 4 (2.6) |
| Current or former | 146 (96.7) |
| Unknown | 1 (0.6) |
| **Stage** |  |
| Limited stage^a^ | 133 (88.1) |
| Extensive stage | 18 (11.9) |
| **Location of surgery** |  |
| Primary tumor | 139 (92.2) |
| Brain metastasis | 7 (4.6) |
| Lymph node | 4 (2.6) |
| Pleural dissemination | 1 (0.6) |

Data are expressed as the median (range) or *N* (%).

^a^ ‘Limited stage’ is defined as a disease confined to one hemithorax, the ipsilateral supraclavicular fossa, or both.


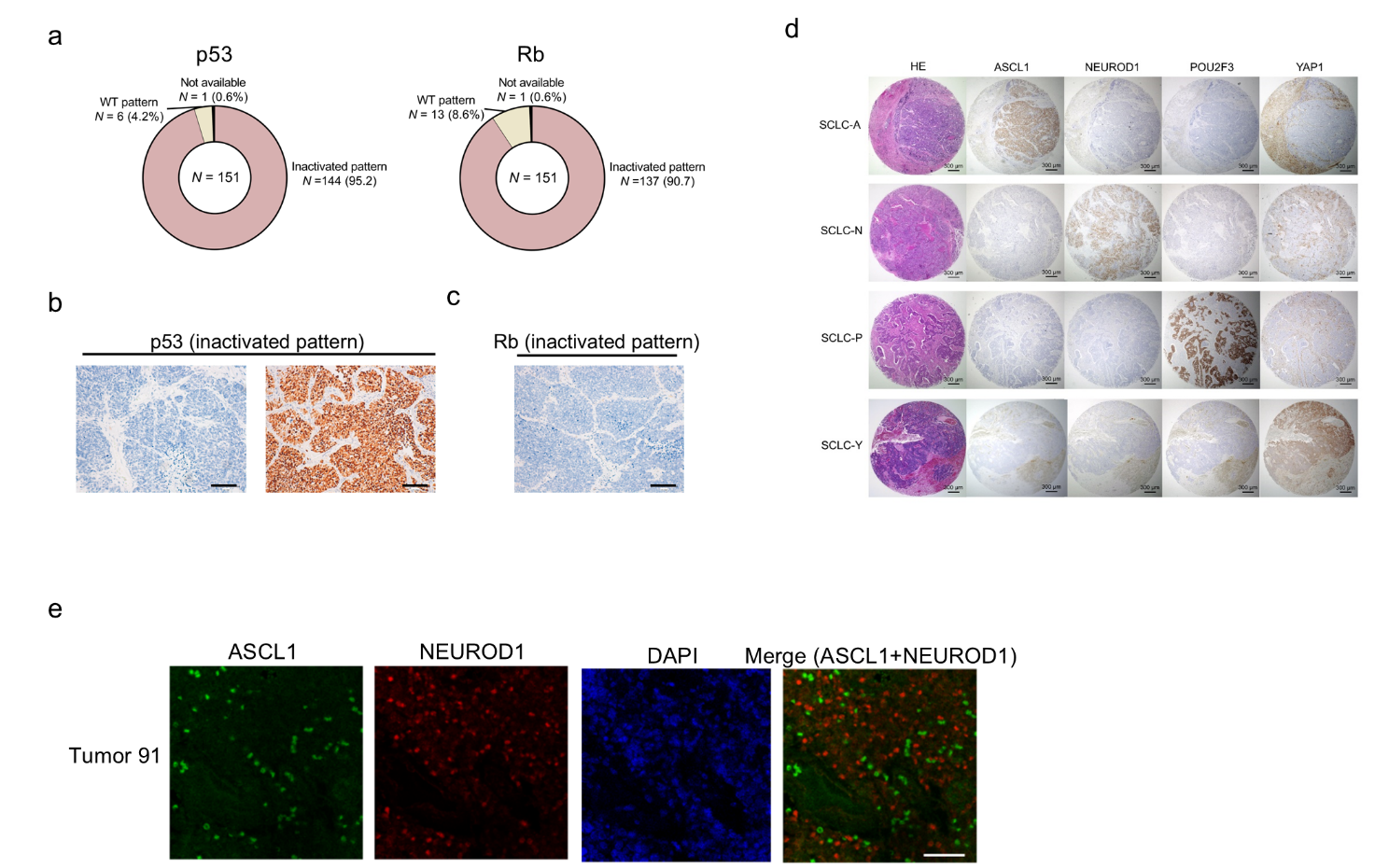


**Supplementary Figure 1. Expression of p53, Rb, and lineage-specific transcription factors in clinical SCLC tumors. (a)** Frequencies of p53 and Rb inactivation in SCLC tumors. Circle charts show the proportions of cases based on the p53 (left) and Rb (right) status. **(b and c)** Representative images of patterns of p53 and Rb inactivation. The complete absence pattern and the strong diffuse staining pattern indicate inactivation of p53 (**b**). Complete absence indicates inactivation of Rb (**c**). Scale bars, 100 µm. **(d)** Representative images of ASCL1-dominant (SCLC-A), NEUROD1-dominant (SCLC-N), POU2F3-dominant (SCLC-P), and YAP1-dominant (SCLC-Y) SCLC tumors. Immunohistochemical procedures were performed on consecutive tissue microarray sections. Scale bars, 300 µm. HE, hematoxylin–eosin. **(e)** Representative images of multiplex immunofluorescence staining of ASCL1 and NEUROD1 in Tumor 91. Scale bar, 50 µm.

**
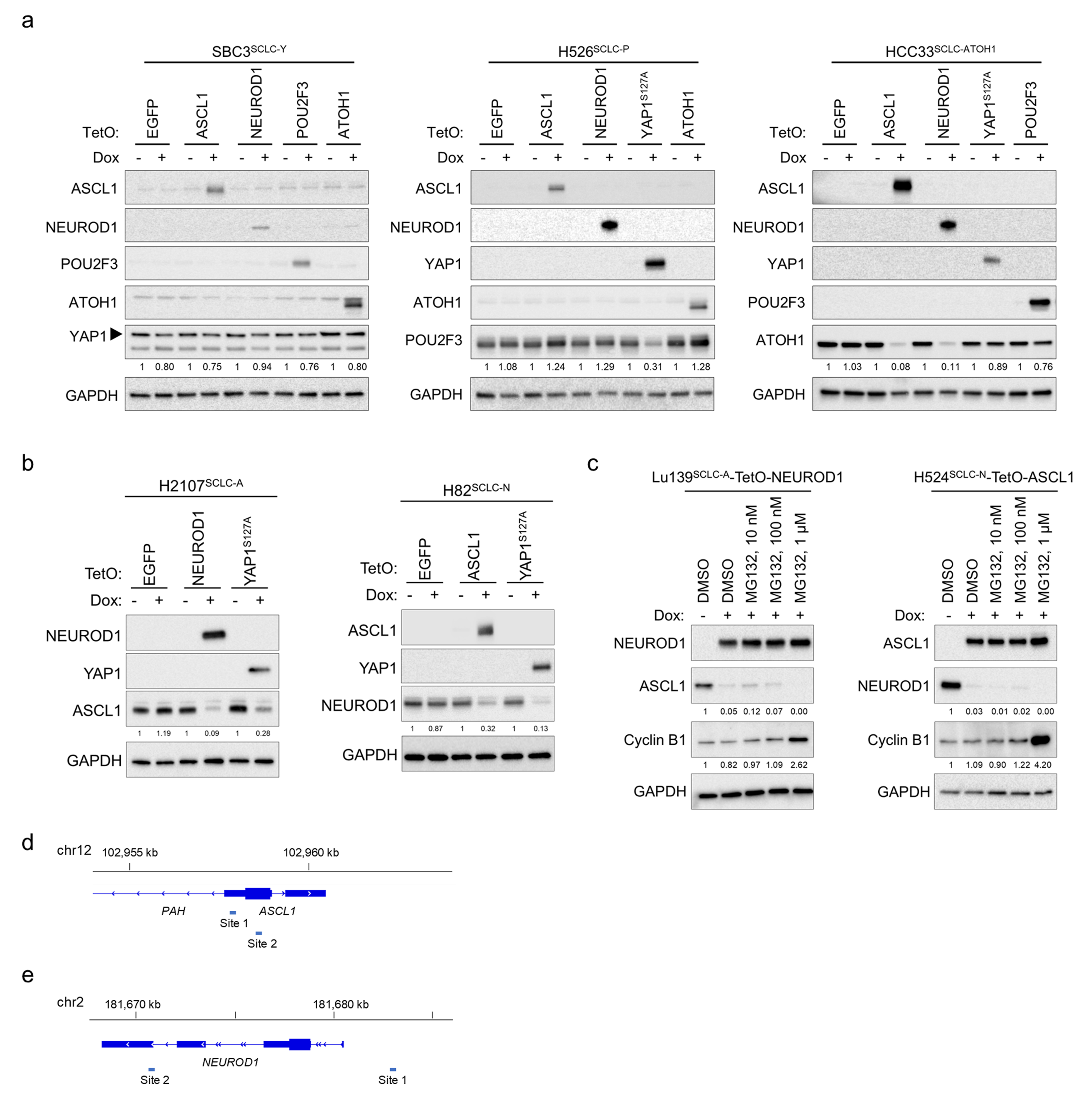
**

**Supplementary Figure 2. Establishment of co-expression models of the lineage-specific transcription factors (TFs) in human SCLC cell lines. (a)** Immunoblots showing the expression of the lineage-specific TFs in SBC3^SCLC-Y^ (left), H526^SCLC-P^ (middle), and HCC33^SCLC-ATOH1^ (right) derivatives before and 5 days after doxycycline (Dox)-mediated induction of other lineage-specific TFs, including ASCL1, NEUROD1, YAP1^S127A^, POU2F3, and ATOH1. Enhanced green fluorescent protein (EGFP) cells were used as controls. The numbers below the YAP1, POU2F3, and ATOH1 blots indicate the values of the bands relative to the corresponding non-doxycycline control values after normalization against GAPDH. **(b)** Immunoblots showing changes in the expression of endogenous ASCL1 in H2107^SCLC-A^ (left) and NEUROD1 in H82^SCLC-N^ (right) derivatives before and 5 days after co-expression of NEUROD1 or YAP1^S127A^ with ASCL1 in H2107^SCLC-A^ cells, and of ASCL1 or YAP1^S127A^ with NEUROD1 in H82^S^^CLC-N^ cells. EGFP cells were used as controls. The numbers below the ASCL1 and NEUROD1 blots indicate the values of the bands relative to the corresponding non-doxycycline control values after normalization against GAPDH. **(c)** Immunoblots showing the expression of exogenous NEUROD1 and endogenous ASCL1 expression following treatment with doxycycline and a proteasome inhibitor, MG132, for 72 hours in Lu139^SCLC-A^-TetO-NEUROD1 cells (left). Immunoblots showing exogenous ASCL1 and endogenous NEUROD1 expression under the same treatment condition in H524^SCLC-N^-TetO-ASCL1 cells (right). Cyclin B1 serves as a positive control to confirm proteasome inhibition by MG132. The numbers below the ASCL1, NEUROD1, and Cyclin B1 blots indicate the values of the bands relative to the DMSO and non-doxycycline control values after normalization against GAPDH. **(d and e)** Target sites 1 and 2 analyzed in the CUT&RUN-quantitative PCR assay within the *ASCL1* **(d)** and *NEUROD1* **(e)** loci are indicated. Each immunoblot image is a representative of at least two independent procedures **(a–c)**.

**
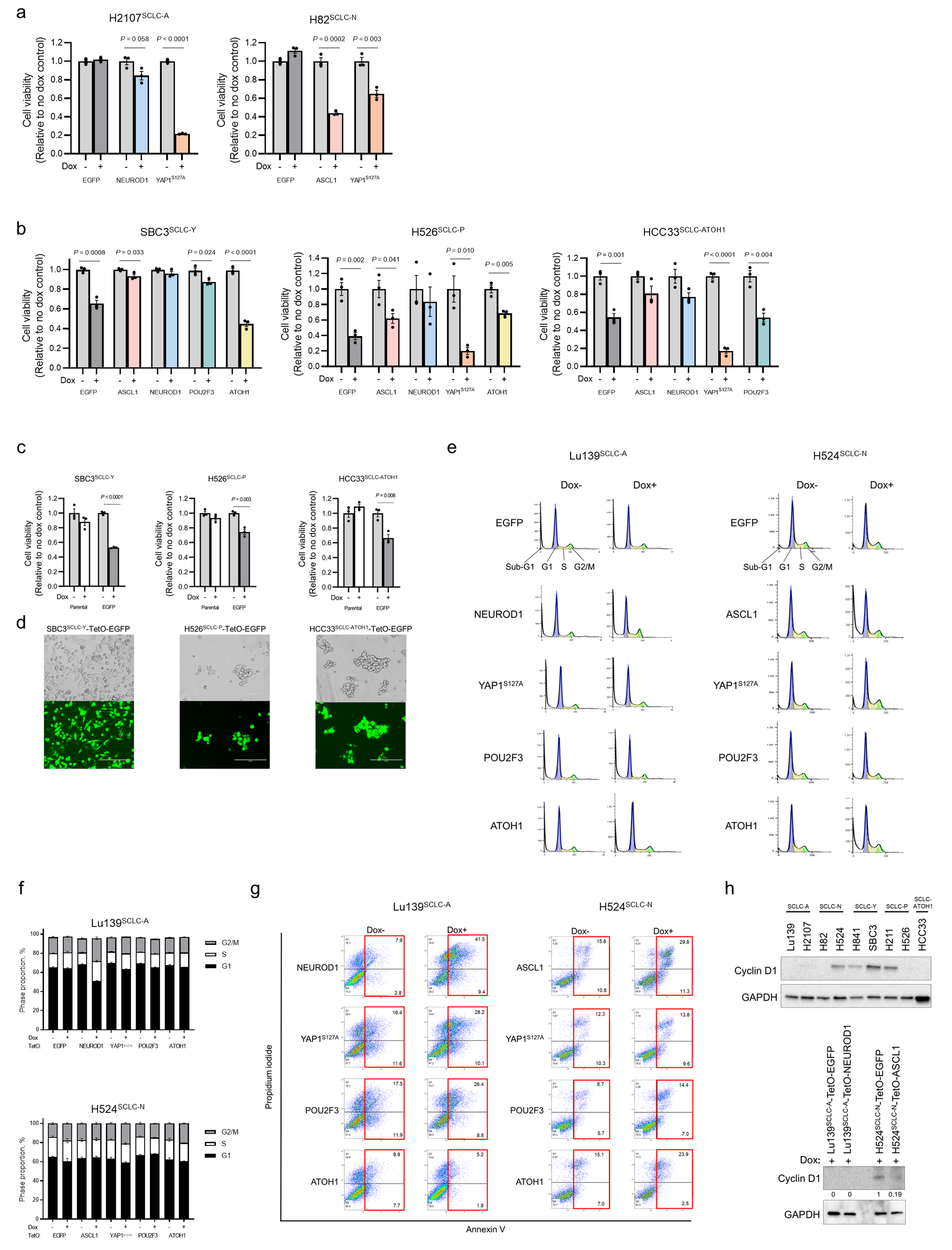
**

**Supplementary Figure 3. Phenotypic effects of the co-expression of lineage-specific transcription factors (TFs) in SCLC cell lines. (a)** Quantification of cell viability relative to corresponding non-doxycycline control cells in H2107^SCLC-A^ (left) and H82^SCLC-N^ (right) derivatives 5 days after co-expression of NEUROD1 or YAP1^S127A^ with ASCL1 in H2107^SCLC-A^ cells, and of ASCL1 or YAP1^S127A^ with NEUROD1 in H82^SCLC-N^ cells. Enhanced green fluorescent protein (EGFP) cells were used as controls. Cell viability was assessed using an alamarBlue cell viability reagent. **(b)** Quantification of cell viability relative to corresponding non-doxycycline control cells in SBC3^SCLC-Y^ (left), H526^SCLC-P^ (middle), and HCC33^SCLC-ATOH1^ (right) derivatives 5 days after co-expression of other lineage-specific TFs. EGFP cells were used as controls. Cell viability was assessed using an alamarBlue cell viability reagent. **(c)** Quantification of cell viability relative to corresponding non-doxycycline control cells in parental SBC3^SCLC-Y^ cells and SBC3^SCLC-Y^-TetO-EGFP cells (left), parental H526^SCLC-P^ cells and H526^SCLC-P^-TetO-EGFP cells (middle), and parental HCC33^SCLC-ATOH1^ cells and HCC33^SCLC-ATOH1^-TetO-EGFP cells (right). Cells were treated with doxycycline for 5 days. **(d)** Photomicrographs (top) and fluorescence phase contrast images (bottom) of EGFP-expressing cells in indicated cell lines. Scale bars, 200 µm. **(e)** Cell cycle assay of Lu139^SCLC-A^ and H524^SCLC-N^ derivatives with or without co-expression of other lineage-specific TFs for 5 days, detected by flow cytometry. **(f)** The cell-cycle phase distributions detected by cell cycle assays after excluding cells in the sub-G_1_ phase in Lu139^SCLC-A^ (top) and H524^SCLC-N^ (bottom) derivatives. (**g**) Representative plots of the apoptosis assay in Lu139^SCLC-A^ (left) and H524^SCLC-N^ (right) derivatives with or without co-expression of other lineage-specific TFs for 5 days. Red squares indicate the area of annexin V-positive cells. **(h)** Immunoblots showing the expression profiles of Cyclin D1 in human SCLC cell lines (top) and immunoblots showing the expression of Cyclin D1 in Lu139^SCLC-A^-TetO-NEUROD1 cells and H524^SCLC-N^-TetO-ASCL1 cells after doxycycline treatment for 5 days (bottom). Corresponding EGFP-expressing cells were used as controls. Each immunoblot image is a representative of at least two independent procedures. Error bars indicate ± the standard error of the mean (SEM) from *N* = 3 independent experiments **(a–c and f)**. Student’s t-test was used for two-group comparisons **(a–c)**.

**
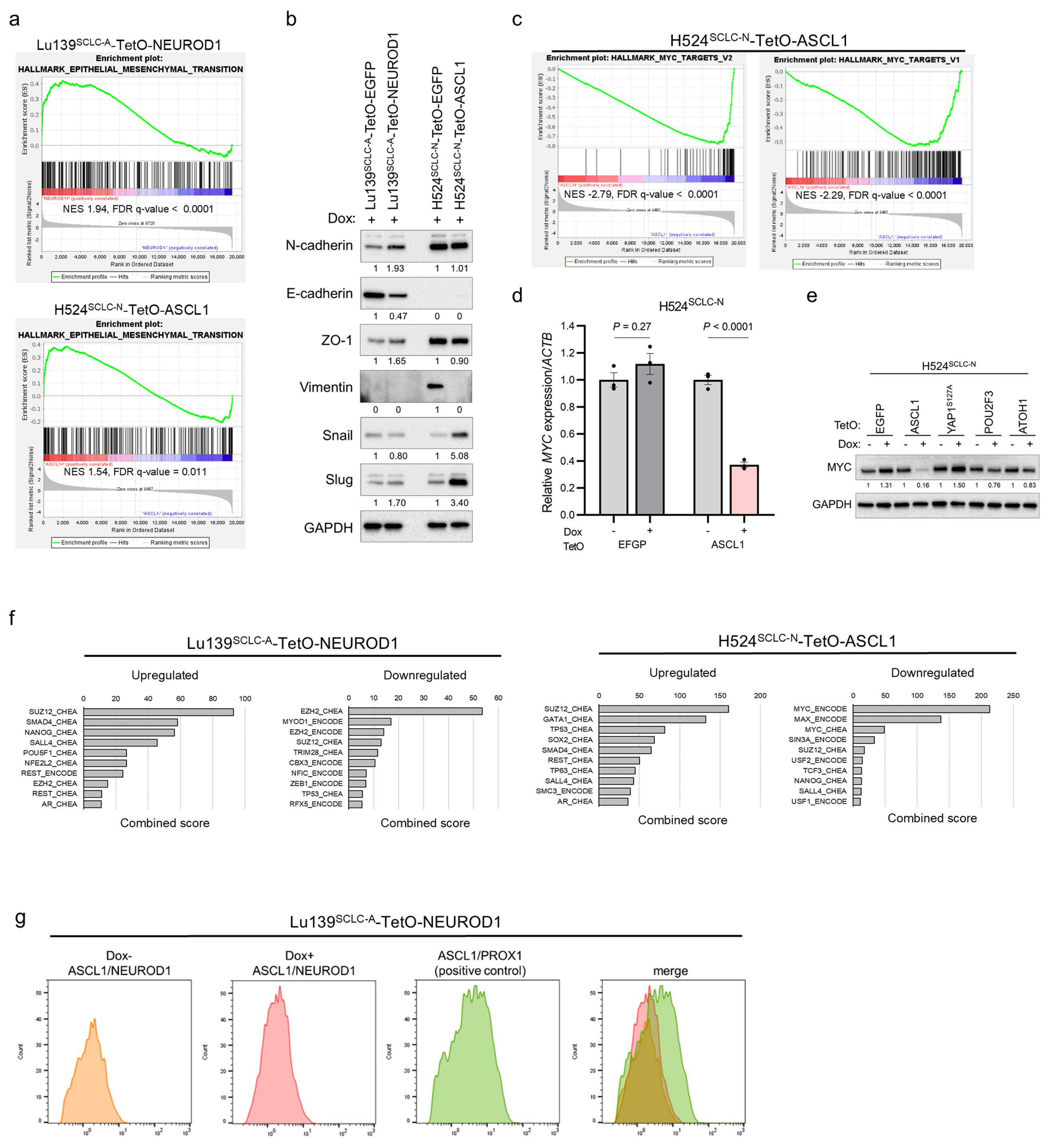
**

**Supplementary Figure 4.** **Gene expression reprogramming following co-expression of ASCL1 and NEUROD1 in Lu139 and H524 cells. (a)** Gene set enrichment analysis (GSEA) plots showing the enrichment patterns of the hallmark epithelial mesenchymal transition gene set in Lu139^SCLC-A^-TetO-NEUROD1 cells (top) and H524^SCLC-N^-TetO-ASCL1 cells (bottom) following doxycycline treatment compared with non-treated controls. NES, normalized enrichment score.  **(b)** Immunoblots showing the expression of epithelial–mesenchymal transition-related proteins in Lu139^SCLC-A^-TetO-NEUROD1 cells and H524^SCLC-N^-TetO-ASCL1 cells after treatment with doxycycline (Dox) for 5 days. Corresponding enhanced green fluorescent protein (EGFP)-expressing cells were used as controls. The numbers below the blots indicate the values of the bands relative to the corresponding EGFP-expressing control values after normalization against GAPDH. **(c)** GSEA plots showing the gene enrichment patterns of hallmark gene sets of MYC target genes in H524^SCLC-N^-TetO-ASCL1 cells compared with non-doxycycline-treated controls. NES, normalized enrichment score. **(d)** Expression levels of *MYC* transcripts after ASCL1 co-expression with NEUROD1 in H524^SCLC-N^ cells relative to the non-doxycycline control after normalization against *ACTB* expression. EGFP-expressing cells were used as a control. Cells were treated with doxycycline for 72 hours. **(e)** Immunoblots showing MYC expression in H524^SCLC-N^ derivatives before and 5 days after the doxycycline-mediated induction of ASCL1, YAP1^S127A^, POU2F3, or ATOH1. The numbers below the MYC blots indicate the values of the bands relative to the corresponding non-doxycycline control values after normalization against GAPDH. **(f)** The top ten candidate factors identified by Enrichr analysis using the significantly upregulated or downregulated genes in Lu139^SCLC-A^-TetO-NEUROD1 cells (left) and H524^SCLC-N^-TetO-ASCL1 cells (right). (**g**) Proximity ligation assay assessing the direct interaction between endogenous ASCL1 and exogenous NEUROD1 in Lu139^SCLC-A^-TetO-NEUROD1 cells with or without doxycycline treatment for 72 hours. PROX1 was used as a positive control for a direct interaction with ASCL1. Error bars indicate ± the standard error of the mean (SEM) from *N* = 3 independent experiments **(d)**. Student’s t-test was used for two-group comparisons **(d)**. Each immunoblot image is a representative of at least two independent procedures **(b and e)**.

**
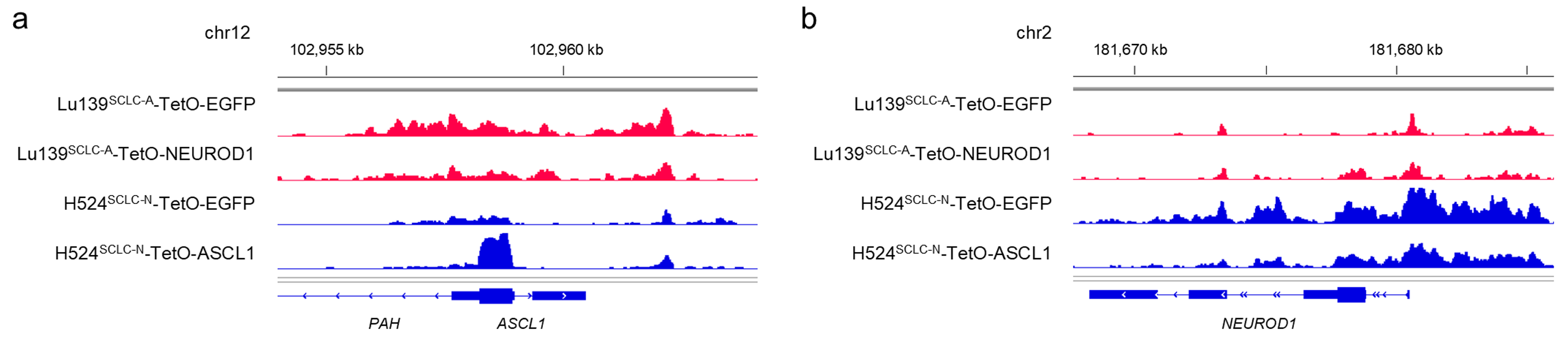
**

**Supplementary Figure 5. Representative tracks of ATAC-seq (assay for transposase-accessible chromatin with sequencing) data at the *ASCL1* (a) and *NEUROD1* (b) gene loci in Lu139^SCLC-A^ cells expressing EGFP or NEUROD1 with ASCL1 (red) and H524^SCLC-N^ cells expressing EGFP or ASCL1 with NEUROD1 (blue).**

**Western blot original data**

**
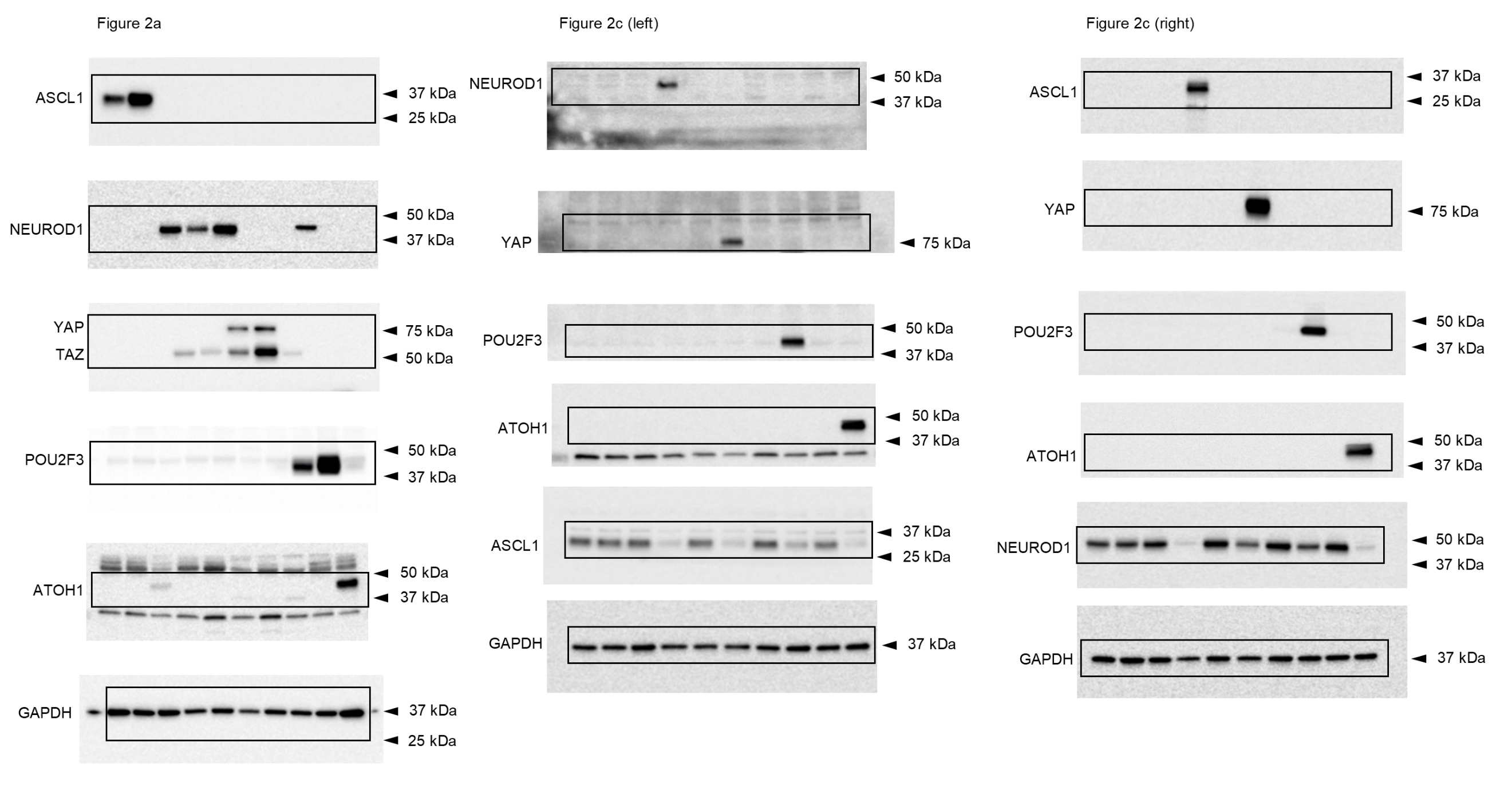
**

**
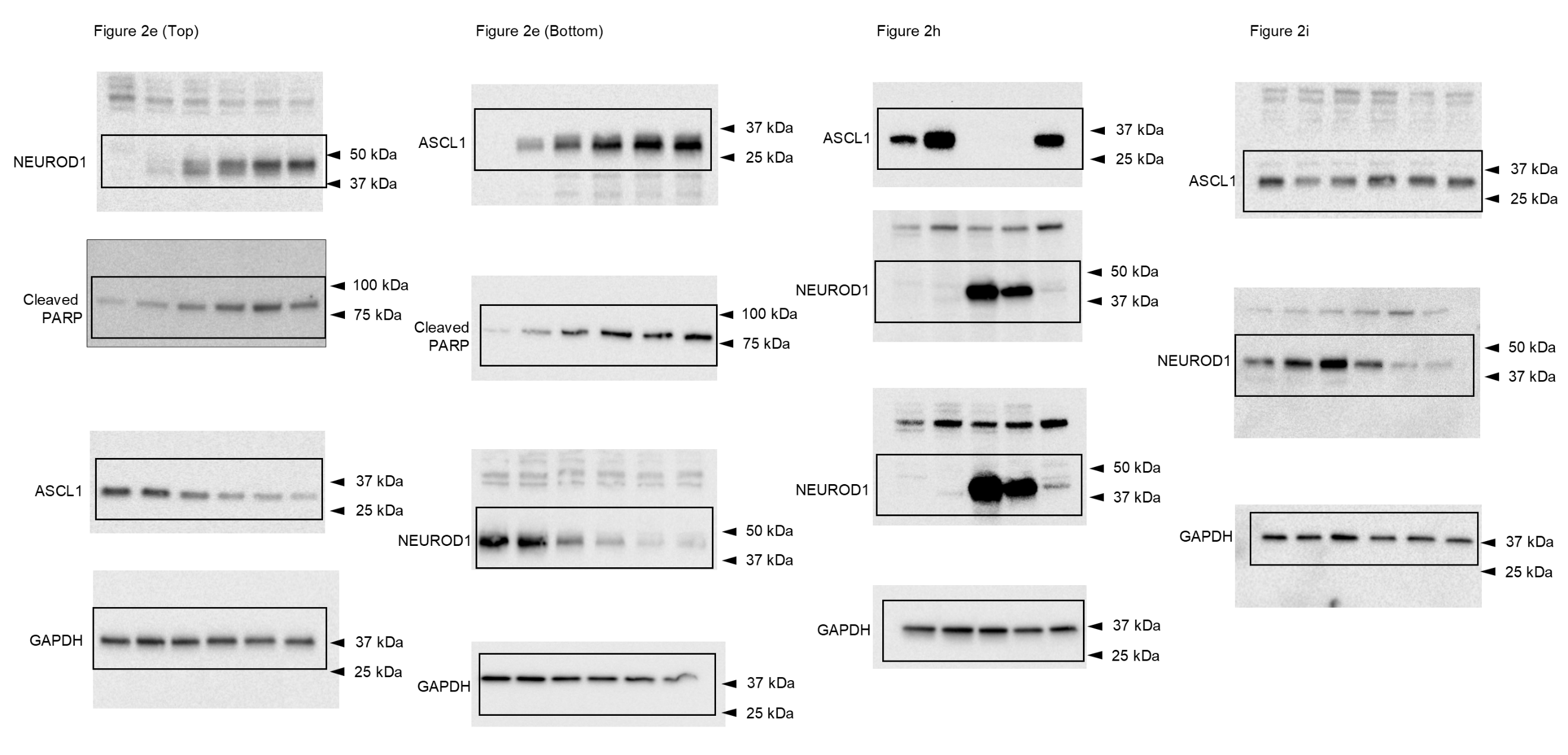
**

**
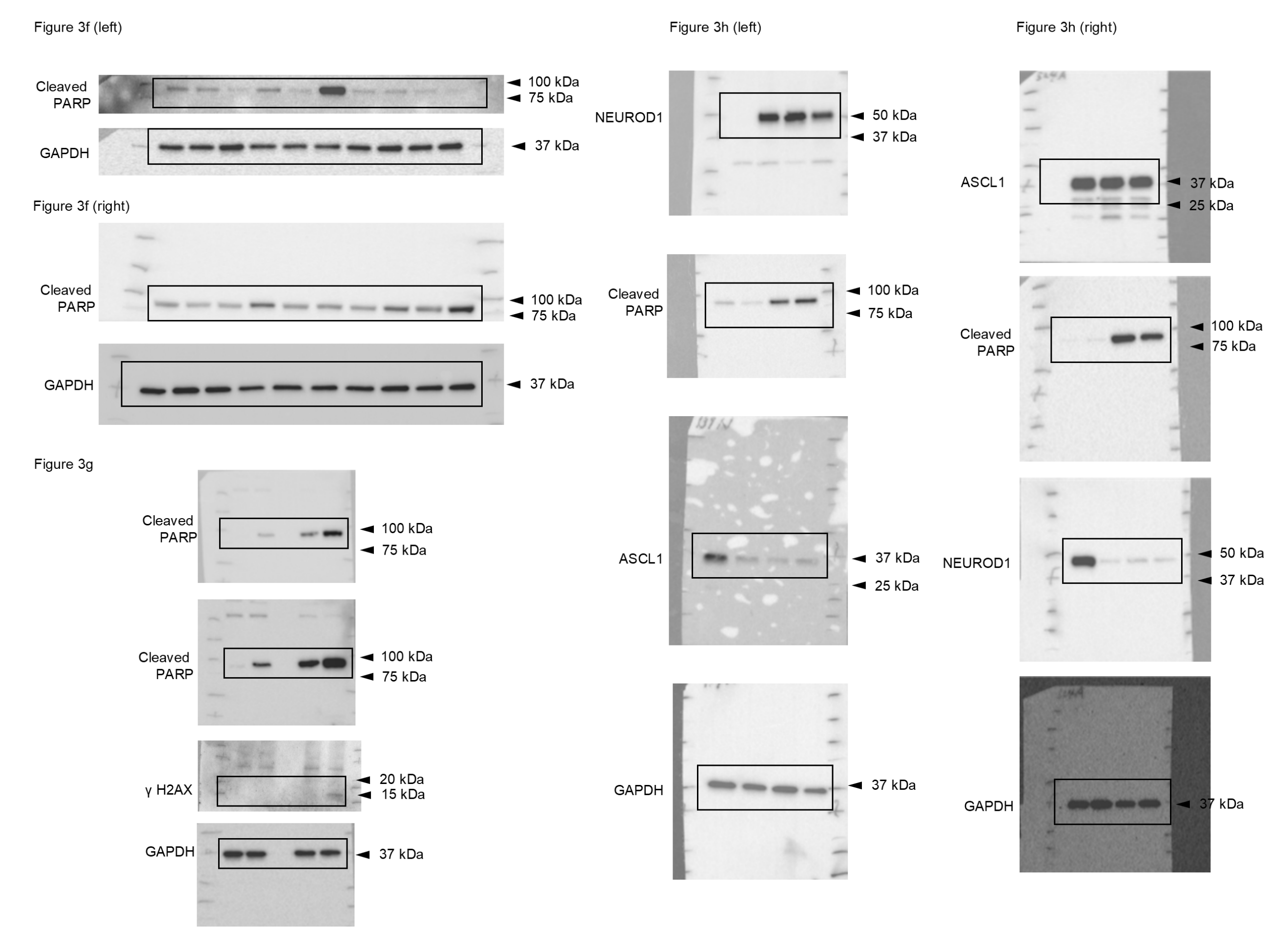
**

**
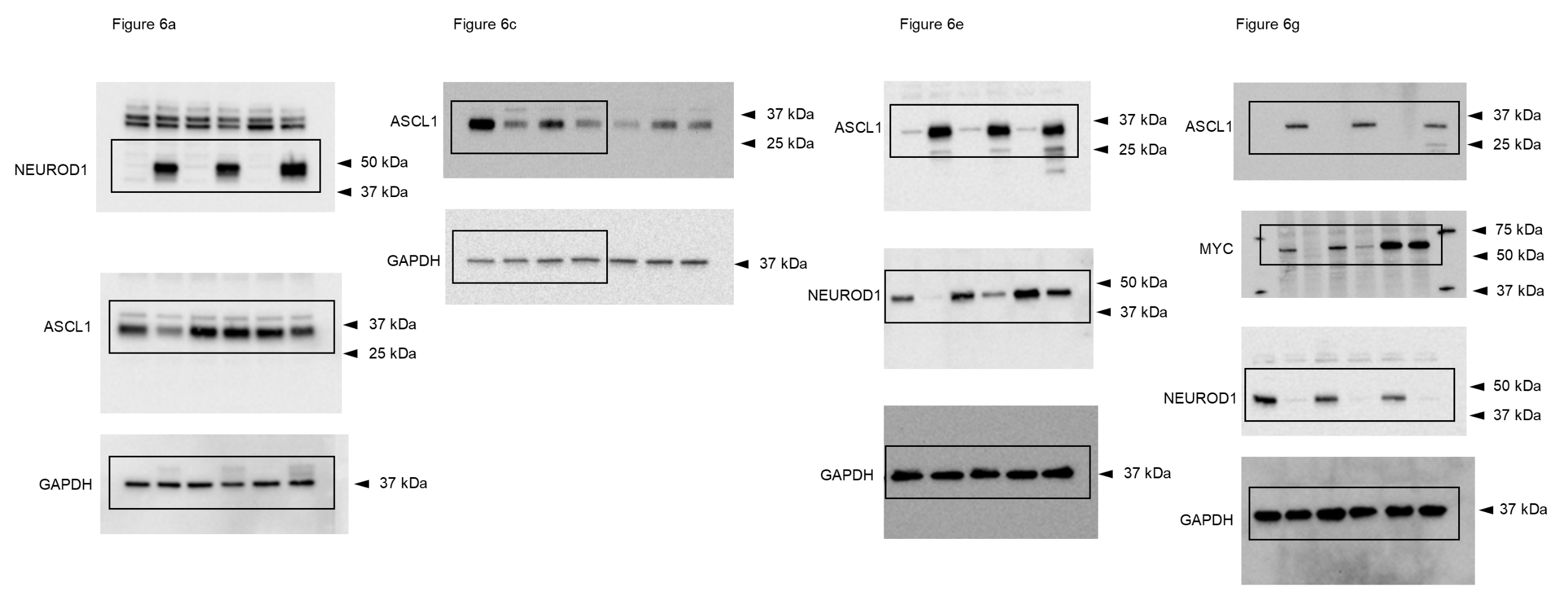
**

**
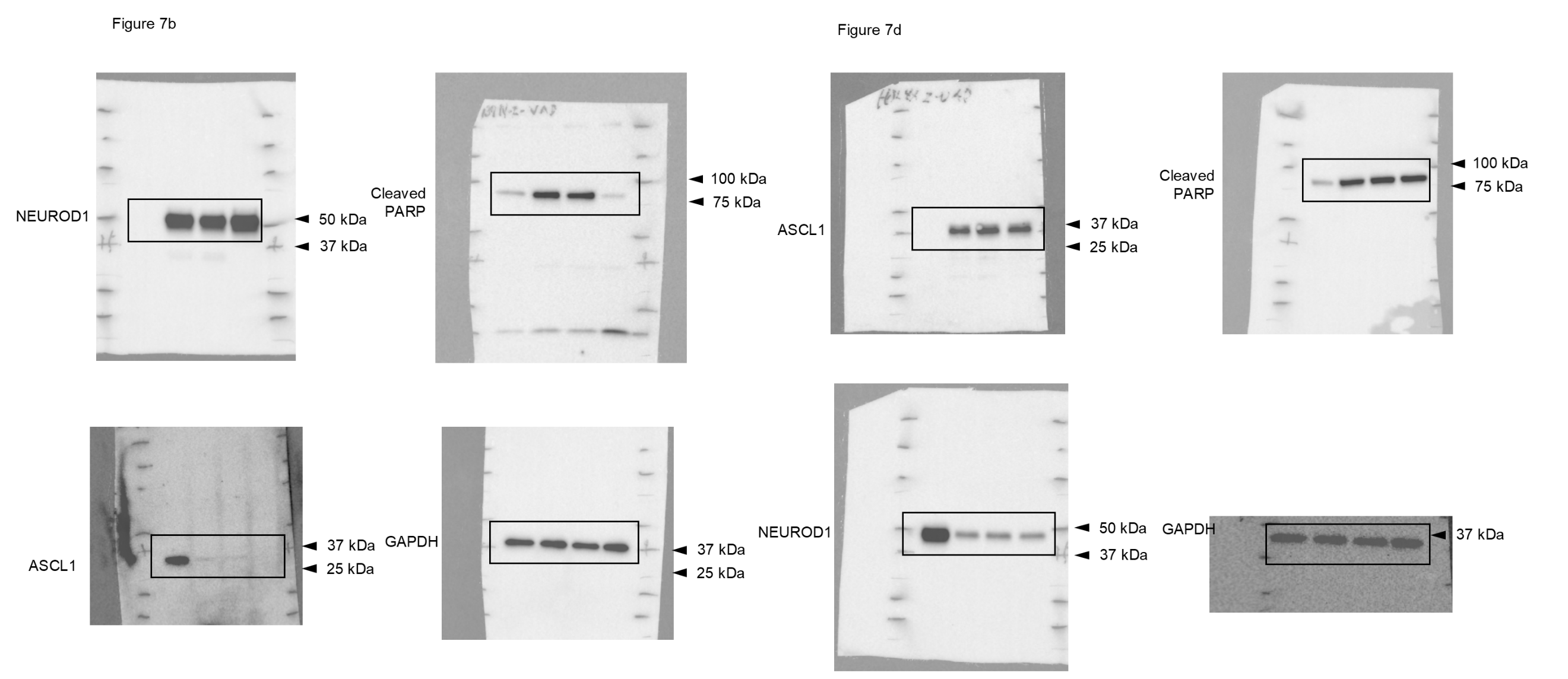
**

**
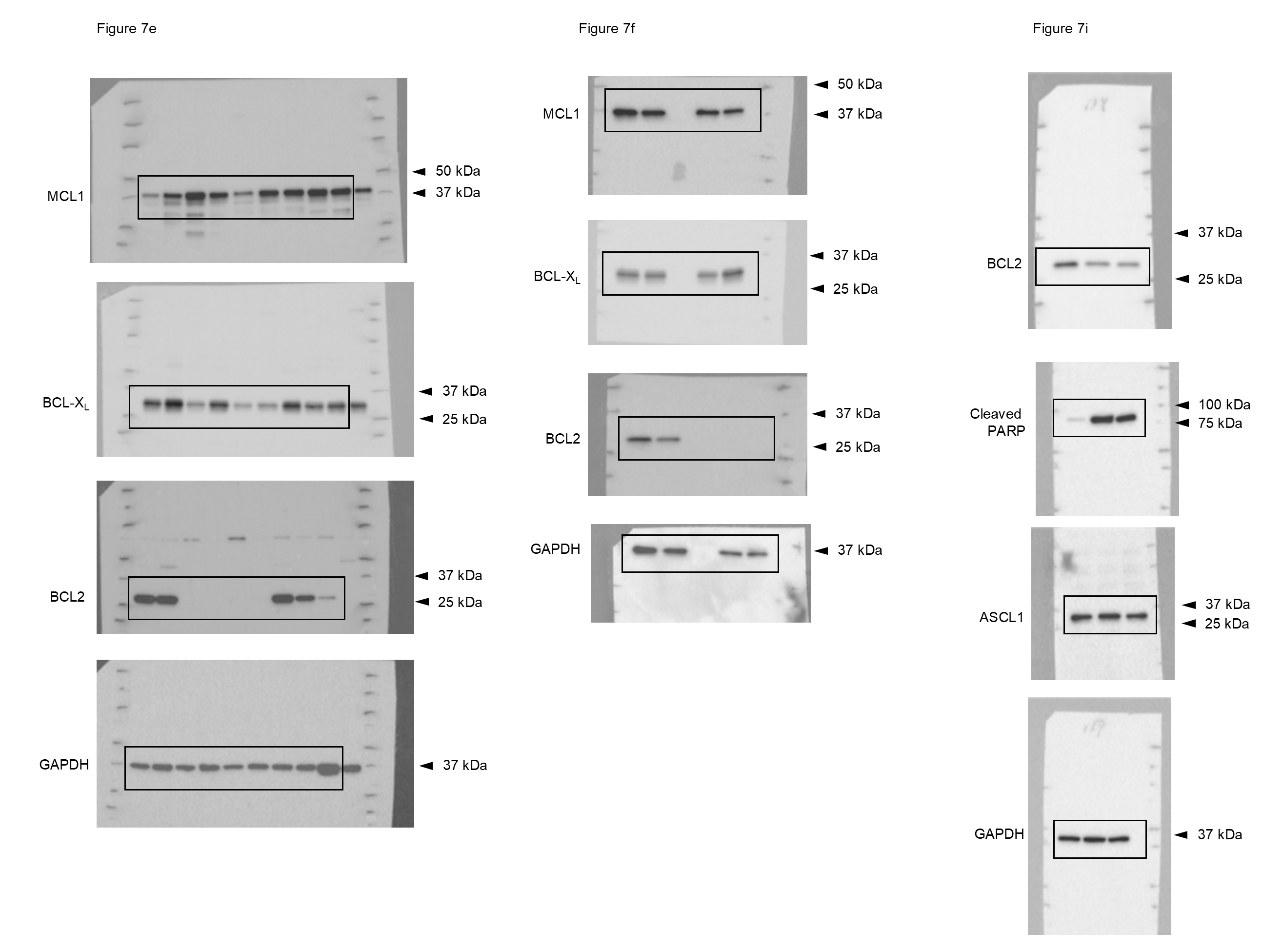
**

**
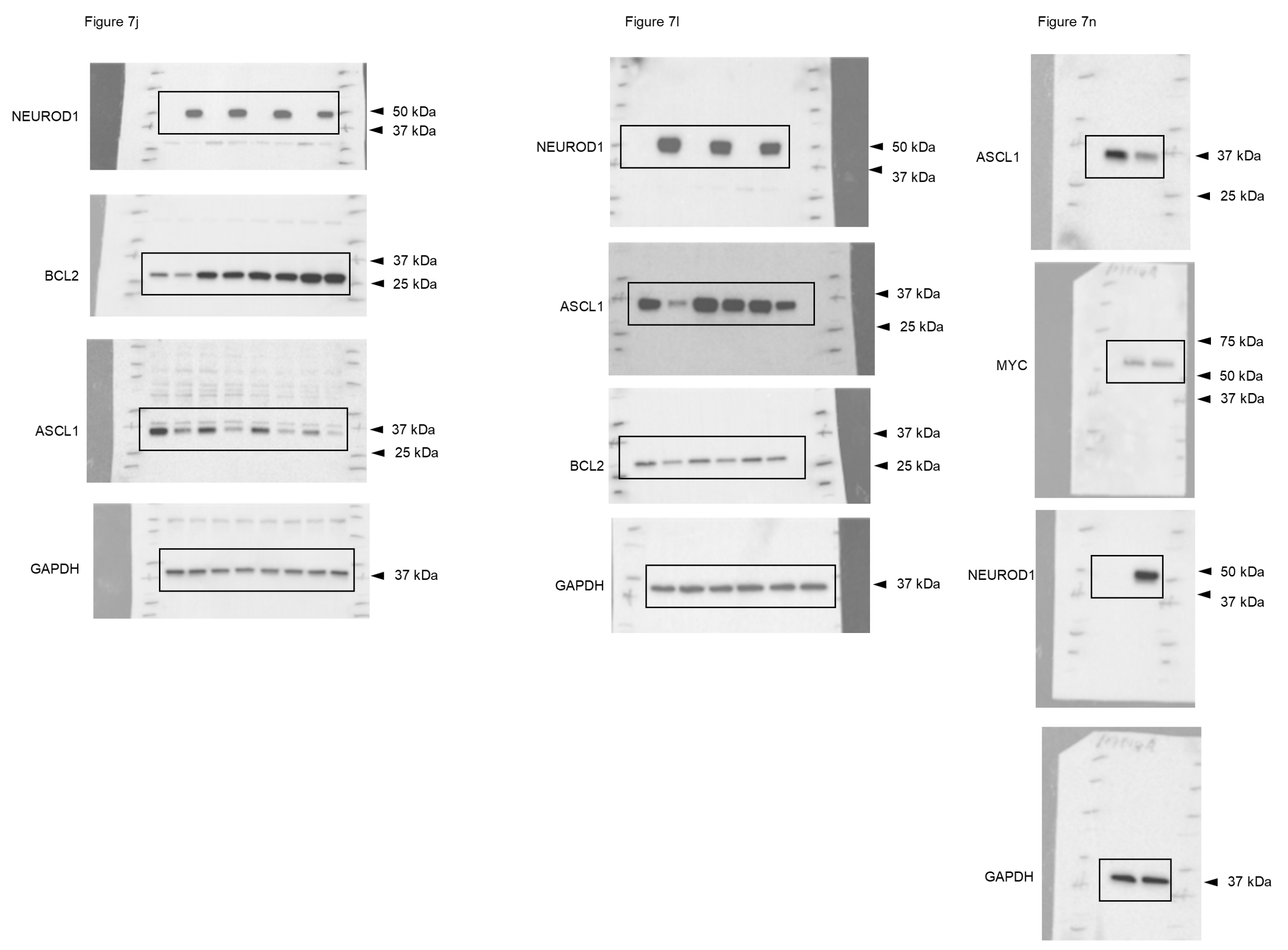
**

**
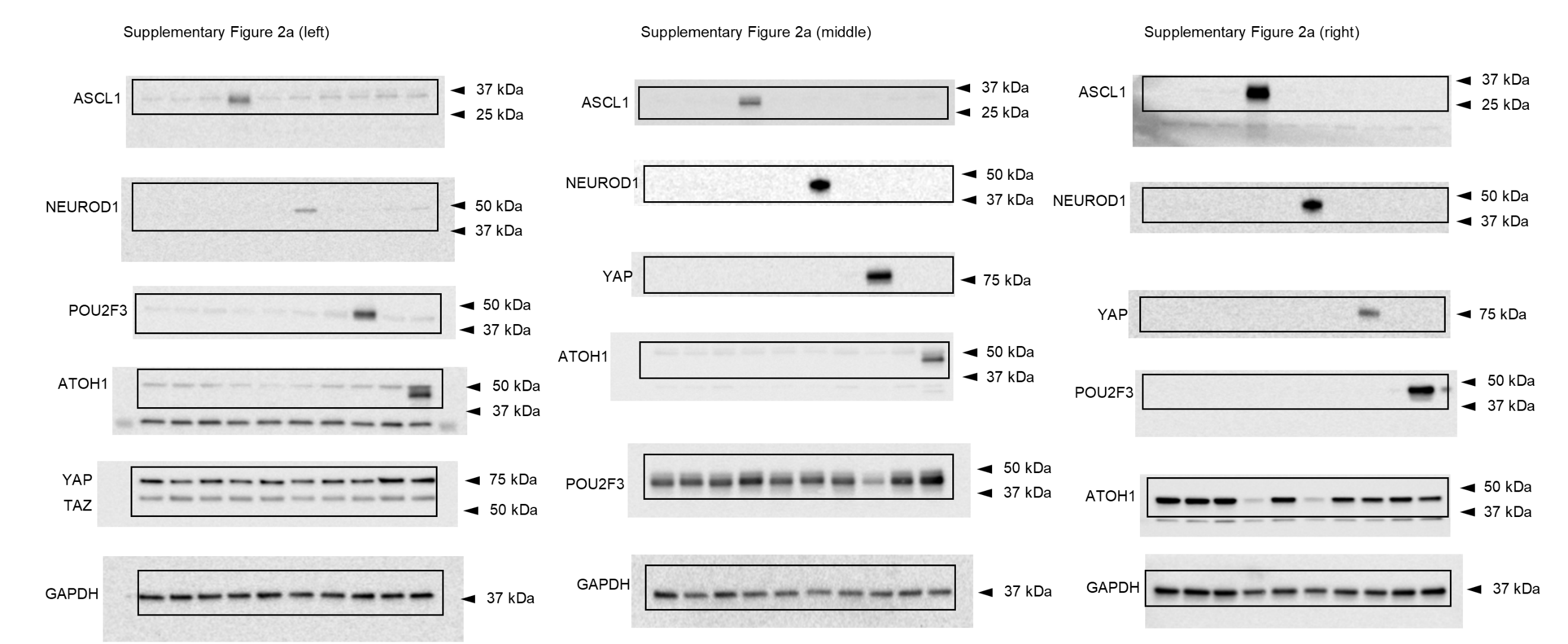
**

**
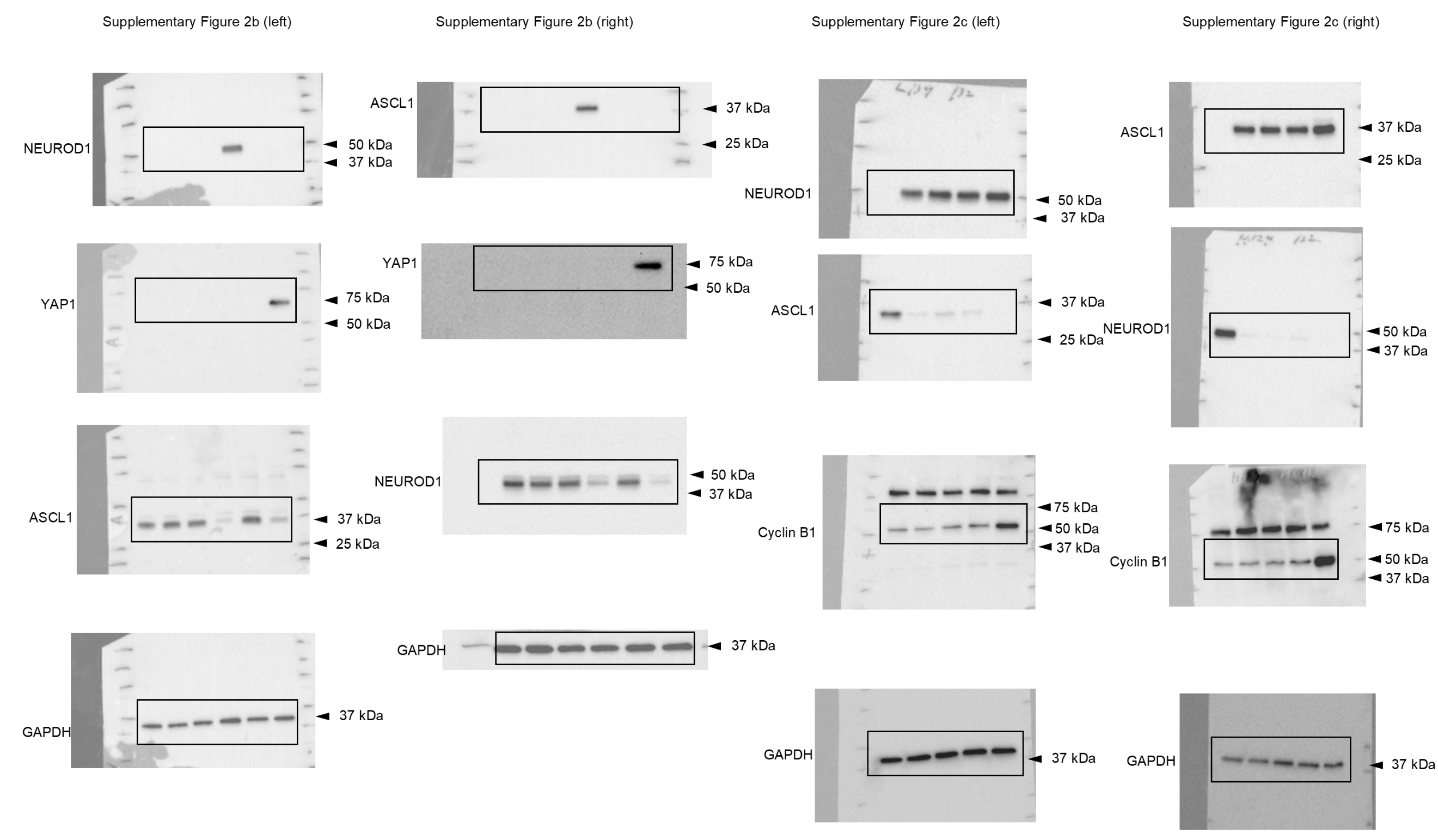
**

**
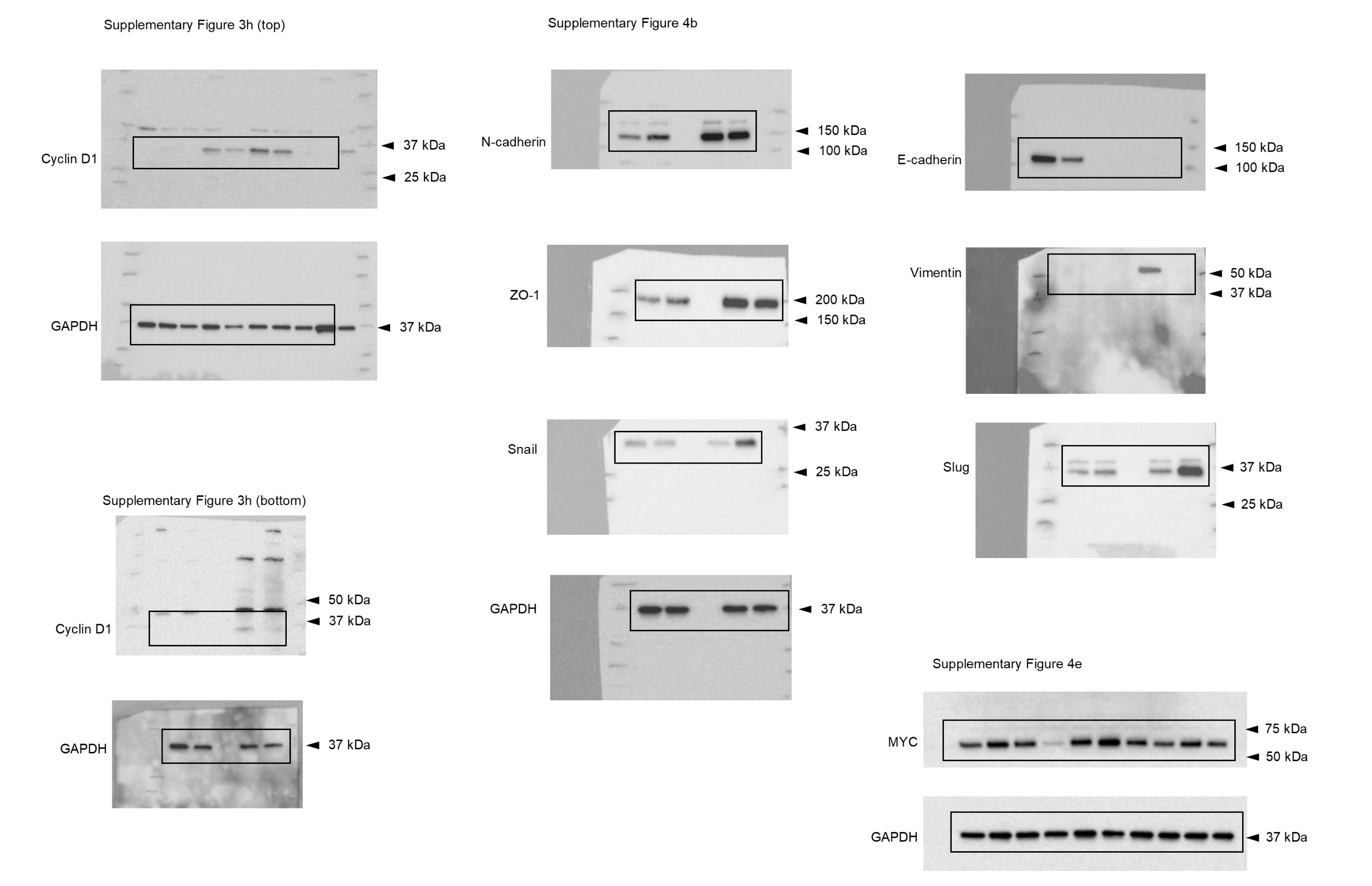
**
